# Shigatoxin encoding Bacteriophage ϕ24_B_ modulates bacterial metabolism to raise antimicrobial tolerance

**DOI:** 10.1038/srep40424

**Published:** 2017-01-20

**Authors:** G. S. Holt, J. K. Lodge, A. J. McCarthy, A. K. Graham, G. Young, S. H. Bridge, A. K. Brown, M. Veses-Garcia, C. V. Lanyon, A. Sails, H. E. Allison, D. L. Smith

**Affiliations:** 1 Faculty of Health and Life Sciences, Northumbria University, Newcastle upon Tyne, UK; 2 Microbiology Research Group, Institute of Integrative Biology, University of Liverpool, Liverpool, UK; 3 School of Medicine, Pharmacy and Health, Durham University, Durham, UK; 4 Public Health England, Royal Victoria Hospital, Newcastle upon Tyne, UK

## Abstract

How temperate bacteriophages play a role in microbial infection and disease progression is not fully understood. They do this in part by carrying genes that promote positive evolutionary selection for the lysogen. Using Biolog phenotype microarrays and comparative metabolite profiling we demonstrate the impact of the well-characterised Shiga toxin-prophage ϕ24_B_ on its *Escherichia coli* host MC1061. As a lysogen, the prophage alters the bacterial physiology by increasing the rates of respiration and cell proliferation. This is the first reported study detailing phage-mediated control of the *E. coli* biotin and fatty acid synthesis that is rate limiting to cell growth. Through ϕ24_B_ conversion the lysogen also gains increased antimicrobial tolerance to chloroxylenol and 8-hydroxyquinoline. Distinct metabolite profiles discriminate between MC1061 and the ϕ24_B_ lysogen in standard culture, and when treated with 2 antimicrobials. This is also the first reported use of metabolite profiling to characterise the physiological impact of lysogeny under antimicrobial pressure. We propose that temperate phages do not need to carry antimicrobial resistance genes to play a significant role in tolerance to antimicrobials.

Colonisation by Shiga toxin-encoding *Escherichia coli* (STEC) causes a potentially fatal gastrointestinal infection in humans. There are currently >500 different characterised STEC serogroups that cause disease including O157:H7 and more recently O104:H4^1^,^2^. Symptoms of STEC infection can vary from bloody diarrhoea through to haemorrhagic colitis, thrombotic thrombocytopaenic purpura (TTP) and haemolytic-uraemic syndrome (HUS), the latter can be fatal^3^,^4^. STEC is a zoonotic infection transmitted from an animal reservoir via contaminated food or water^5^. Shiga toxins are responsible for the severe downstream sequelae due to high cytotoxicity to human renal microvascular endothelial cells^4^,^6^. Importantly, the genes encoding Shiga toxin (Stx) are disseminated by temperate bacteriophages.

Stx-phages enter one of two replication pathways: a productive lytic life cycle or a more passive lysogenic cycle where the prophage is replicated by the bacterium as any other genetic loci. The lytic-lysogen decision of lambdoid-like bacteriophages is regulated by early gene expression and sequential binding of proteins to a well characterised genetic switch^7^,^8^,^9^.

The co-evolutionary interaction between a phage and its bacterial host is dynamic, with interplay linked to rounds of inhibition, selection and evolution, often referred to as an ‘arms race’^10^. Smith *et al*.^11^ used a multi-loci PCR typing approach to demonstrate that no two Stx phage isolates had the same genotype^11^. This heterogeneity is further supported by Bonanno *et al*.^12^ who identified multiple Stx-phage morphologies not previously reported^12^. Stx-phages are closely related to bacteriophage lambda, with a comparable genome organisation. In comparison to lambda, Stx-phages carry significantly larger amounts of DNA (~20–25 Kbp), with up to 73% of the genome and putative coding genes having no known function when analysed at either the nucleotide or protein level^13^. Nevertheless these genes are well conserved across many Stx-phages and thus likely to be important to the biology of the phage or its bacterial host^13^. Upon phage infection and conversion to a lysogen, genes that are accessory to the core biology of the phage may offer a selective advantage to the host. Mis-excision, mis-packaging of phage DNA^14^, and recombination^15^ play a large role in phage genome variation. This may leave phage DNA regions, remnant or cryptic prophages, that positively impact on the selection and survival of both the phage and the bacterium^16^. This is further supported by the common occurrence of prophage regions, usually multiple, in the chromosomes of many bacterial pathogens^17^.

There are a number of features of the Shigatoxigenic phage vB_EcoPϕ24_B_ or ϕ24_B_ that are particularly relevant to the success and persistence of Stx prophages in *E. coli*. In contrast to the lambda infection model, ϕ24_B_ can multiply infect a bacterial host^18^,^19^,^20^. Stx-phages have been isolated from a wide variety of environments where *E. coli* is present, and this has undoubtedly been promoted by the use of an essential outer membrane protein BamA as the adsorption site^21^. This interaction is conserved in Stx-phages as the incidence of the tail and host recognition protein is widespread^21^. ϕ24_B_ has also been shown to survive well in compost models^22^, and infectivity after 30 days in bovine manure and slurry^23^. ϕ24_B_ is genetically similar to phages isolated from sporadic outbreaks of STEC with high virulence and therefore a good model of the viruses circulating in *E. coli* populations in the environment^13^.

The ability of lambdoid like phages to increase virulence by carriage of toxins in their accessory genome^24^ is well described e.g. the cholera toxin (CTX) carried by *Vibrio cholera* phage^25^,^26^. In Stx-phage genomes the shigatoxin genes are always located at the same position, upstream of the Q antiterminator gene therefore organisation and gene location is important^27^. It has been hypothesised that presence of *stx* also offers selection and stability for the lysogens^28^. Colon *et al*.^29^ observed that Stx-prophages show greater levels of spontaneous induction than lambda but this more readily correlates to Rec dependent and independent control of the CI repressor protein rather than presence or absence of *stx*^29^.

There are other accessory genes that are seemingly superfluous to viral replication have been shown to aid microbial selection against environmental stress. Examples include: antibiotic resistance^30^,^31^, acid tolerance^32^,^33^ and polylysogeny^34^. Phage gene expression has also been shown to aid adhesion and colonisation, for example the expression of the λ-encoded lom gene promotes adhesion to buccal epithelial cells^35^, and the λ-encoded bor confers serum resistance^36^. Other phage encoded virulence traits include exotoxin production in *E. coli*^37^ and increase in bacterial invasion via *Staphylococcus* phage encoded kinase that influences fibrinolysis^38^. Bacteriophage ϕ24_B_ has also been shown to encode a mi-RNA in the *lom* region that alters expression of anti-repressor *d-ant* and downstream activity of CI, leading to rapid induction^39^. Tree *et al*.^40^ identified 55 prophage regions encoding small regulatory RNA within the Sakai *E. coli* O157:H7 strain^40^. These small prophage anti-sRNA had the ability to form complexes or mimic core genome regulatory sRNA to aid selective advantage to the bacterial host in bovine rectal mucus. Stx-phage ϕ24_B_ shows 98% sequence homology at the nucleotide level to the remnant Stx2 phage present in the Sakai genome^13^.

The function of the large numbers of hypothetical proteins encoded by ϕ24_B_ and other converting phage is difficult to determine, and a focus of this study, as gene expression or interaction may be specific to an environment or subject to selective pressure. Therefore, current approaches *in vitro* using synchronous cultures and standard laboratory conditions to investigate the role of these prophages are challenging. The function of these hypothetical gene products and how they impact the host in either an advantageous or deleterious way may be missed. In this study we focus on phage mediated antimicrobial tolerance to antibiotics found in the livestock farm setting which is the primary reservoir of pathogenic shigatoxigenic *E. coli*.

Our principal aim was to identify how infection and integration of ϕ24_B_ changes the microbial physiology, and how phage conversion aids selection compared to its naïve counterpart. This study demonstrates an increase in cell proliferation in standard culture, increased respiration activity using the Biolog phenotypic array and antimicrobial tolerance that is not linked to an identifiable resistance gene-cassette. The Biolog phenotypic array is a good tool for rapidly identifying phenotypic changes in the respiration profiles of bacteria^41^,^42^. This study also presents an untargeted metabolomics approach to reveal key phage-mediated differences in metabolism directly linked to biotin and fatty acid synthesis. Interestingly, we show that integration of ϕ24_B_ into its primary integration site located 250 bp upstream of the IntS gene^43^ allows converted *E. coli* MC1061 to grow using alternative sources of phosphate compared to the naïve bacterial host. We show for the first time unique changes in bacterial metabolite profiles on phage conversion in response to sub-inhibitory concentrations of antimicrobial agents.

## Results

### ϕ24_B_ integration increases cell proliferation

**Figure 1:**
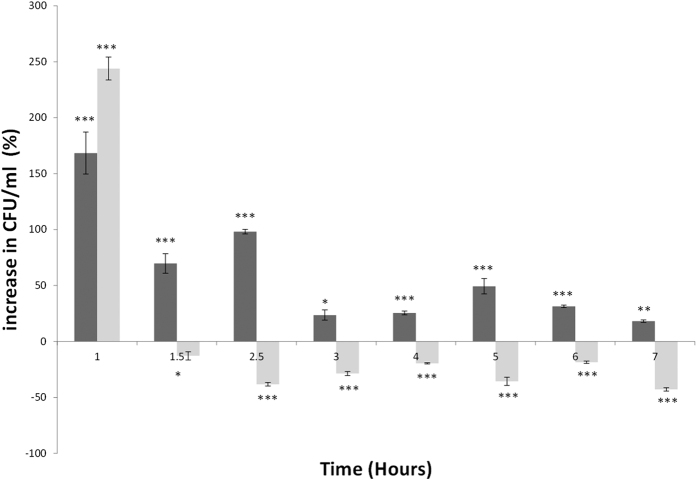
Clustered column graph representing percentage increase in cell proliferation of single (ϕ24_B_::ΔKan, dark grey) and double (ϕ24_B_::ΔKan, ϕ24_B_::ΔCat, light grey) MC1061 lysogens. Cultures were grown at 37 °C (CFU.ml) and samples taken over a 7 hour period including experimental and technical replicates (n = 9). Percentage increases or decreases show differences in growth of the lysogens compared to the uninfected MC1061 represented here as 0 on the x axis. Significance threshold *P* values ***<0.001, **<0.01, *<0.05, significance below the x axis demonstrates greater growth from the Naïve host.

Growth rates of bacteria can differ due to a range of environmental parameters. To investigate the impact of ϕ24_B_ on *E. coli*, viable cell counts were determined during growth comparing *E. coli* B strain MC1061 to single and double lysogens, the latter integrated into separate locations in the MC1061 chromosome^43^. Under standard growth conditions, the single and double lysogens showed significantly higher cell numbers compared to the naïve MC1061 (>200%, Fig. 1). This alongside a statistically significant increase in doubling time of 18 minutes for the single lysogen compared to 20 minutes for the naïve MC1061 (*p* < 0.006), calculated from each growth curve (data not shown, n = 9). As the cultures reached mid to late exponential growth, the differences in growth rates diminished (Fig. 1). The greatest difference in growth was identified in early growth (Fig. 1). This was supported by a shorter lag time in the single and double lysogen compared to MC1061 with a 0.5 and 1.8-fold increase respectively in cell number after the first hour of growth. Stationary phase in the double lysogen is achieved earlier compared to MC1061 and the single lysogen as nutrients are utilised rapidly alongside the accumulation of inhibitory components of growth.

### ϕ24_B_ integration alters utilisation of different mono-phosphates and inability to respire using β-D-Allose

To explore the single lysogen related differences in cell respiration during growth we used the Biolog Phenotype MicroArray. This determined functional changes in respiration and metabolism resulting from phage conversion over a 48 h period with recordings taken every 15 min. The lysogen acquired the ability to respire and grow utilising uridine-2-monophosphate (U-2-P) when compared to the naïve MC1061 (SI Fig. 1, panel A). Phage mediated subversion of pyrimidine and purine synthesis by lytic phages has previously been reported and will be discussed later. Conversely, integration of the phage inhibited the lysogens ability to use D-Allose for respiration.

### ϕ24_B_ integration alters resistance to osmotic stress or antimicrobials

**Figure 2:**
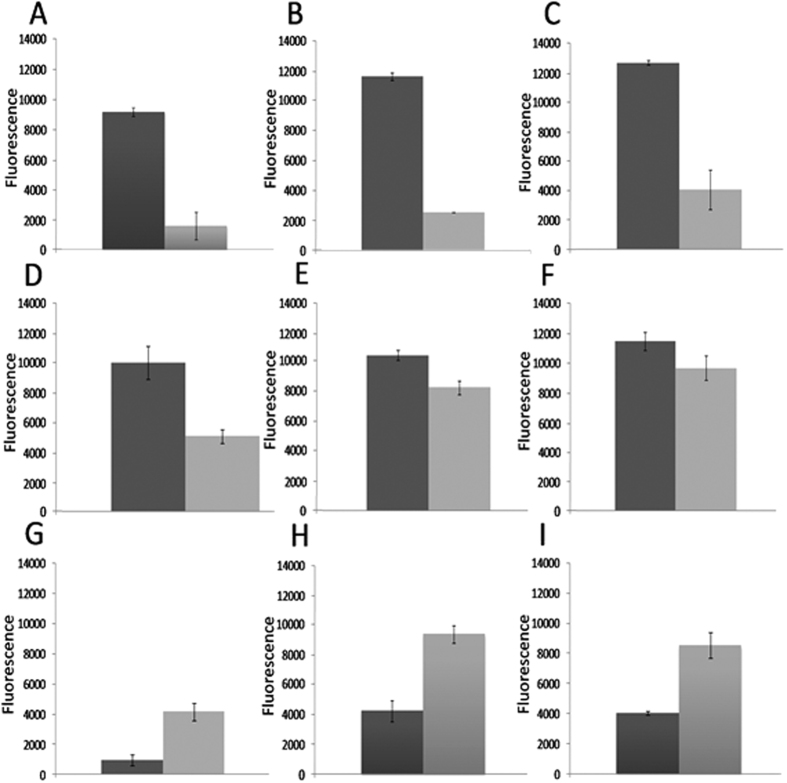
A comparison of Area Under the Respiration Curve (AURC) data from the Biolog bacterial phenotypic microarray. Data plotted shows the addition of supplemented nutrients or chemical challenge showed statistically significant difference in rates of respiration between the lysogen and naïve MC1061 host (for *P* values see SI Table 2). Arbitrary Omnilog fluorescence values (y-axis) show differences between the naïve MC1061 (light grey) host and the lysogen (dark grey) over a 47.5 h time period (n = 3). Error bars represent SEM. Graphs A-F show significantly higher amount of respiration of the lysogen compared to the naïve host under the following conditions; (**A**) U-2-monophosphate, (**B**) 8-hydroxyquinoline, (**C**) chloroxylenol, (**D**) cefoxitin, (**E**) cefomendole and (**F**) amoxacillin. Graphs (**G**–**I**) show mean AURC values where growth on different carbon sources or chemical challenge that has a detrimental effect on the respiration of MC1061 when converted by ϕ24_B_, these inculde; (**G**) β_D-Allose, (**H**) ofloxacin and (**I**) oxolinic acid.

Again using the Biolog phenotypic array the single lysogen is able to tolerate a range of antimicrobial agents that have both extracellular and intracellular targets (Fig. 2). The respiration curves derived for this experiment are provided in the supplementary information (SI - Figs 1 and 2). Tests showing differences in respiration profile were determined in the presence of 22 antimicrobials and 7 increases in salt concentration (SI Table 2). Of these 29 different tests, the lysogen showed a level of tolerance to 17 antimicrobials (SI Table 2). Data presented in Fig. 2 (n = 3) are comparisons of the area under the respiration curve illustrating those that were altered significantly. ϕ24_B_ infection promotes tolerance to 8-hydroxyquinoline (*P* < 0.000), chloroxylenol (*P* < 0.0037), and cefmetazole (*P* < 0.0026), cefoxitin, (*P* < 0.015) cefemendole (*P* < 0.0239) and amoxicillin (*P* < 0.057). Integration of ϕ24_B_ into the primary site 250 bp upstream of *IntS* inhibits respiration utilising B-D-allose. Lysogeny also limits cell respiration in the presence of oxolinic acid although this is linked to phage induction as the cellular target is DNA gyrase. Inhibition of DNA gyrase has been previously shown to stimulate temperate phages to the lytic life cycle as cellular stress stimulates RecA, LexA and proteolytic cleavage of the repressor protein promoting phage induction^44^.

### ϕ24B integration increases MC1061 tolerance to sub-inhibitory concentrations of chloroxylenol and 8-hydroxyquinoline

**Figure 3:**
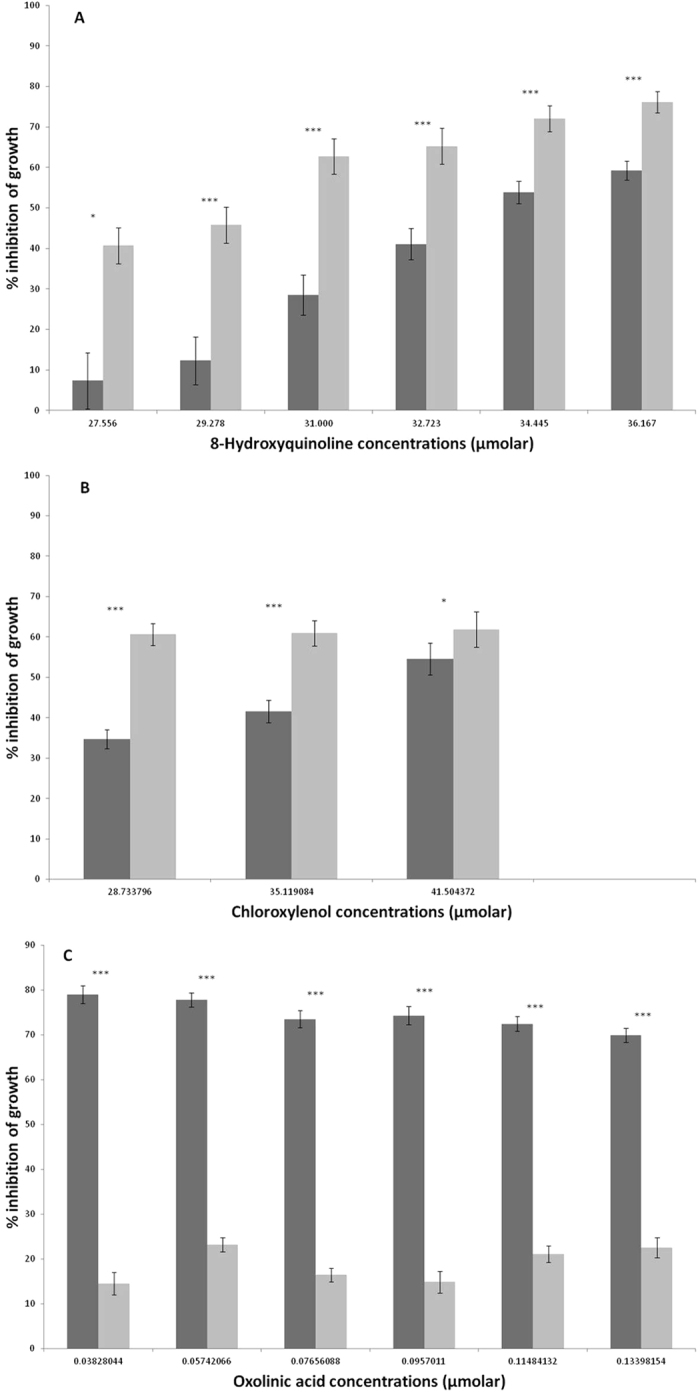
Response in growth of both MC1061 (light grey) and the ϕ24_B_ lysogen (Dark grey) to an increasing concentration of (**A**) 8-hydroxyquinoline, (**B**) chloroxylenol, and (**C**) oxolinic acid. Bacterial growth was measured by increase in optical density at 600 nm after 18 hours growth at 37 °C, as per original Biolog assay. Error bars represent the standard error of the mean (SEM) (n = 12). Significance represented by (*P*) thresholds; ***<0.001, **<0.01, *<0.05.

To better understand the level of antimicrobial tolerance of the single lysogen, we first determined sub-inhibitory concentrations (SIC) against both MC1061 and the lysogen that reduce cell growth by ~60%. The antimicrobials chloroxylenol, oxolinic acid and 8-hydroxyquinoline were selected to validate the Biolog data. Prior to comparison, an approximate SIC range was determined for MC1061 utilising each of the 3 test drugs. Figure 3 illustrates increased tolerance by the lysogen in the presence of chloroxylenol and 8-hydroxyquinoline. Conversely, the naïve host shows increased tolerance compared to the lysogen in the presence of oxolinic acid. This also offers a positive control for the assay as oxolinic acid targets DNA gyrase and therefore stimulates phage induction^26^. Phage induction was confirmed by the presence of free phage compared to the un- induced control (data not shown).

### Metabolic profiles comparing naïve MC1061 to ϕ24_B_ Lysogen

We used an untargeted metabolite profiling approach using high resolution LC-MS (≤1 ppm mass accuracy in full scan) to determine metabolic differences between bacterial host and lysogen during growth and when challenged with a sub-inhibitory concentration of test antibiotic. To broadly compare findings, significant metabolic differences (*p* < 0.05) were observed between both growth phase and antimicrobial challenge. In total, >11 K ion features or possible metabolites were determined across all of the different tests performed. Of these 81 showed discrimination between the naïve MC1061 and the ϕ24_B_ lysogen that had clean chromatogram peaks and <5% coefficient variable (CV) (SI Table 3). These 81 metabolites that show differences can be further stratified to each test.

**Figure 4:**
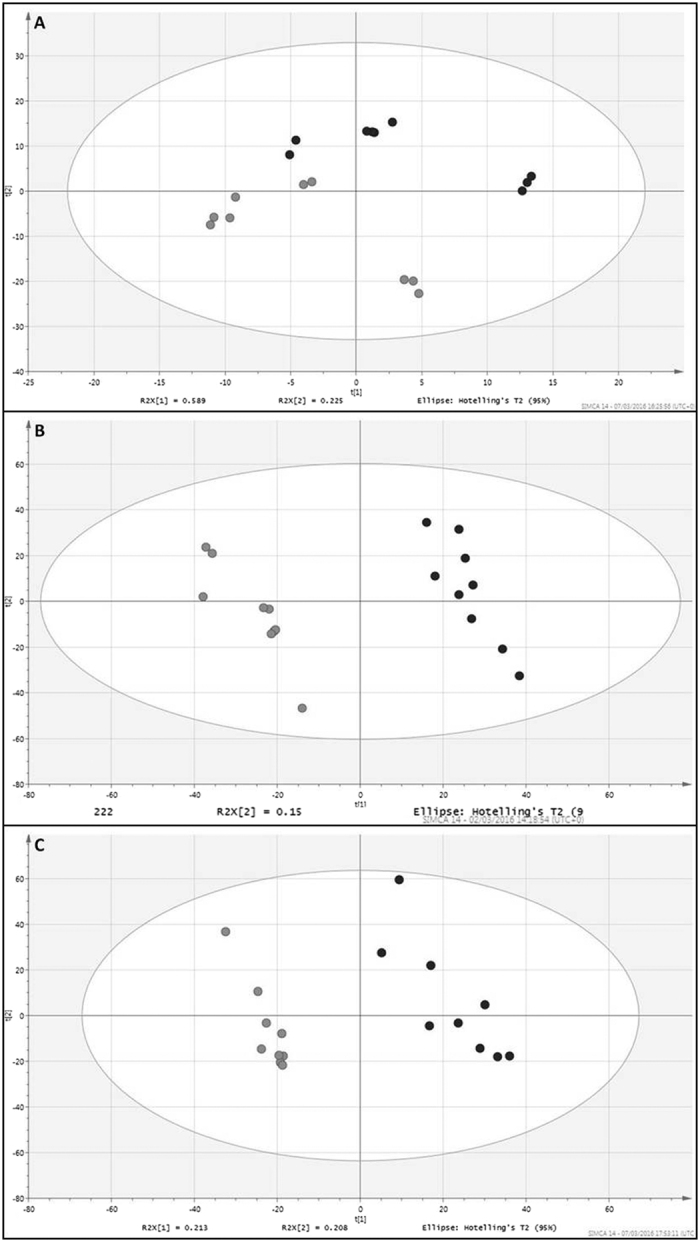
(**A**–**C**) The metabolite profiles of MC1061 versus lysogen and multivariate analysis using partial least discriminant analysis (PLS-DA). The panels represent score plots from PLS-DA models of: (**A**) Standard growth conditions, and supplementation with (**B**) 8-hydroxyquinoline and (**C**) chloroxylenol, between the naïve host (light grey spot) and lysogen (dark grey spot), the model discriminatory parameters for the PLS-DA analysis are described in the results section and in SI Table 5.

The metabolite data was analysed using supervised and non-supervised methods of multivariate analysis. Principal Component Analysis was first employed to visualise trends in the dataset and identify potential outliers. To further interrogate the data, Partial-Least Squared Discriminant Analysis models (PLS-DA) were generated and score plots are shown in (Fig. 4A–C). The PLS-DA models for both hydroxyquinoline and chloroxylenol conditions score plots had good discriminating ability, establishing the metabolic differences between the lysogen and naïve host. During standard growth conditions component 1 failed to discriminate: Q2 −0.556, R2Y 0.262, as R2Y and Q2 < 0.5, although certain metabolites showed significant differences between the lysogen and MC1061. The 8-hydroxyquinoline component 1: Q2 0.74, R2Y 0.89 and the chloroxylenol component 1: Q2 0.802, R2Y 0.923 were both discriminatory with an R2Y and Q2 > 0.5. Further model statistics can be found in the supplementary information SI Table 5. Stx-phage ϕ24_B_ has been previously shown to undergo spontaneous induction^27^ and may impact the metabolite profile through sequestration of host function and movement to lysis. We therefore compared the metabolite profiles of both the lysogen and MC1061 with a phage inducing agent, oxolinic acid (DNA gyrase inhibitor). No correlation was seen between metabolite profiles of the lysogen or MC1061 when compared to that of the lysogen undergoing induction with oxolinic acid (data not shown).

### ϕ24_B_ integration alters the metabolite profile of MC1061 in standard growth conditions

Out of the 81 discriminatory metabolites determined in this study, only 16 were shown to discriminate between the naïve host and single lysogen under standard culture conditions. Of these 16 metabolites 4 were found in higher levels in the lysogen. This suggests that the lysogen down regulates certain metabolic functions or is directing metabolism along a different pathway, or both. It is likely to support the change in biology we report in this work and increased rates of early growth by the lysogen.

Early growth in the lysogen demonstrates an observable difference in metabolic profile compared to the naïve MC1061. Under standard growth conditions during early growth, 5 metabolites in total were shown to discriminate between the naïve MC1061 and lysogen. Of these, 1 was higher compared to the naïve control (Fig. 3 SI). During stationary phase, in standard growth conditions, only 9 metabolites in total showed significant difference and all were found in lower levels in the lysogen.

As phage-mediated metabolic differences are present during standard culture, the differences in metabolite profiles under challenge with sub-inhibitory concentrations of 8-hydroxyquinoline and chloroxylenol were tested (Fig. 2B,C). The previous Biolog results showed that the lysogen displays a tolerance to these 2 antibiotics.

### **ϕ24_B_ integration alters the metabolite profile of MC1061 during growth under sub-inhibitory concentrations of 8-hydroxyquinoline**

Upon treatment with 8-hydroxyquinoline, there were 29 metabolites that showed significant difference between the naïve MC1061 and single lysogen. Of these 29 metabolites, 22 were found in higher levels in the lysogen. Early growth phase in the lysogen demonstrates an observable difference in metabolite profile compared to naïve MC1061. Under 8-hydroxyquinoline stress during early growth, 6 metabolites in total were shown to discriminate between the naïve MC1061 and lysogen. Of these, 5 were higher compared to the naïve control (Fig. 3 SI).

### **ϕ24_B_ integration alters the metabolite profile of MC1061 during growth under sub-inhibitory concentrations of chloroxylenol**

Under chloroxylenol treatment, 41 metabolites showed significant differences between the naïve MC1061 and lysogen. Of these 41, the lysogen had 22 metabolites with significantly higher levels compared to the naïve host. Early growth phase in the lysogen demonstrates an observable difference in metabolic change compared to the naïve MC1061. Under chloroxylenol stress during early growth, 13 metabolites in total were shown to discriminate between the naïve MC1061 and lysogen. Of these, 9 were higher compared to the naïve control (Fig. 3 SI).

### **Alteration in metabolomics profile and antimicrobial tolerance is not linked to kanamycin resistance selective marker used to detoxify ϕ24_B_**

The kanamycin gene (aph3) used to detoxify the ϕ24_B_ phage^18^ is used as a selective marker only prior to experimentation. As there is no consistency in the metabolic profiles when the lysogen cultures are treated with the 2 different antimicrobials this cannot be driven by ϕ24_B_ encoding aph3. The metabolite profiles are also discriminatory to each of the 2 antimicrobials tested.

### Characterising the metabolites that discriminate between the naïve MC1061 and ϕ24_B_ lysogen

The discriminatory metabolites determined from each test were compared with metabolite databases and were putatively identified based on exact mass and empirical formula (LCMS methods section). The identity of each metabolite was confirmed using fragmentation analysis using a secondary MS/MS stage. Identities with fragment similarity were found for 58 of the 81 metabolites discriminating between the naïve and lysogen. We focus here on 6 particular metabolites as they have robust identities from fragmentation patterns, retention times, and low accurate mass error (PPM), relating to known curated bacterial metabolites (Table 4 SI). The 6 metabolites are: hexadecanoic acid (a fatty acid that is utilised in the construction of lipid A), sphinganine (putative kinase), 5-Methyluridine (nucleotide synthesis, specifically pyrimidine), ophthalmic acid (glutathione analogue), pimelic acid and FAPy-Adenine, with PPM error margins of 0 ± 1 (0.17, −0.64, 0.45, 1.31, 0.56 and −1.00, respectively).

**Figure 5:**
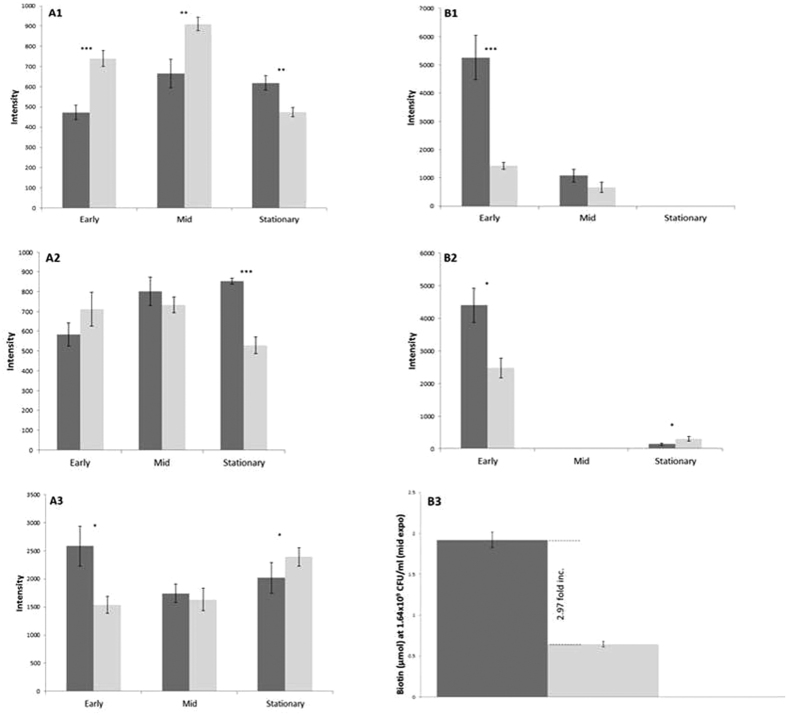
Biotin concentration, FAPy-Adenine and pimelic acid intensity showing significant biological differences between naïve host and lysogen during growth and antimicrobial challenge. (**A1**): Changes in cellular stress marker FAPy-Adenine abundances under the challenge of chloroxylenol at early, mid and stationary growth between the lysogen (dark grey) and naive Host (light grey). (**B1**): Average pimelic acid abundance under chloroxylenol at early, mid and stationary growth between the lysogen and naive Host. (**A2**): Average FAPy-Adenine abundances under selective pressure of 8-hydroxyquinoline at early, mid and stationary growth between the lysogen and naive MC1061. (**B2**): Average pimelic acid abundances under challenge with 8-hydroxyquinoline at early, mid and stationary growth between the lysogen and naive Host. (**A3**): Average pimelic acid abundances under standard conditions at early, mid and stationary growth between the lysogen and naive Host. (**B3**): Variance in the amounts of Biotin present in samples of Ф24_B_ lysogen and MC1061 naïve Host. Error bars derived from standard error of the mean (n = 3). Biotin Quantitation test performed using BioVision® quantitation kit (7.5) using a modified protocol. Two tailed significance represented by ***<0.001, **<0.01, *<0.05, key: *Inc. = Increase, *expo = exponential growth.

The lysogen has significantly higher intensity levels of pimelic acid under all tests, specifically during early growth (Fig. 5). FAPy-Adenine, a bacterial stress marker^45^, is only seen in stressed conditions in these analyses, with the lysogen expressing significantly lower intensity during early growth and higher intensity at stationary phase growth (Fig. 5). Hexadecanoic acid is identified in significantly higher abundance under cellular stress of chloroxylenol, and is further increased in the lysogen during early growth (*P* = 0.04). Metabolite sphinganine is present under standard conditions in higher intensity in the naïve MC1061. When challenged with chloroxylenol, intensity levels of sphinganine were undetectable in both naïve and lysogen during early growth. During mid-exponential and stationary phase growth under choloroxylenol test there is >100 fold increase in intensity of sphinganine in both the naïve and lysogen. 5-Methyluridine is present at stationary phase in all conditions, and is also identified in higher intensity when challenged with both antibiotics. Ophthalmic acid was present at all stages of growth under standard conditions where the lysogen shows lower intensity at early and mid-growth, and higher levels at stationary phase. When treated with either antimicrobial agent, ophthalmic acid was only present at stationary growth, with significantly higher intensity found in the lysogen (*P* = 0.001). During standard culture, there are 16 metabolites responsible for the differences seen between the core metabolic profiles of naïve host and lysogen during the 3 growth phases. Importantly 10 of these, including pimelic acid, are also present when the lysogen is challenged with chloroxylenol and 8-hydroxyquinoline.

**Figure 6:**
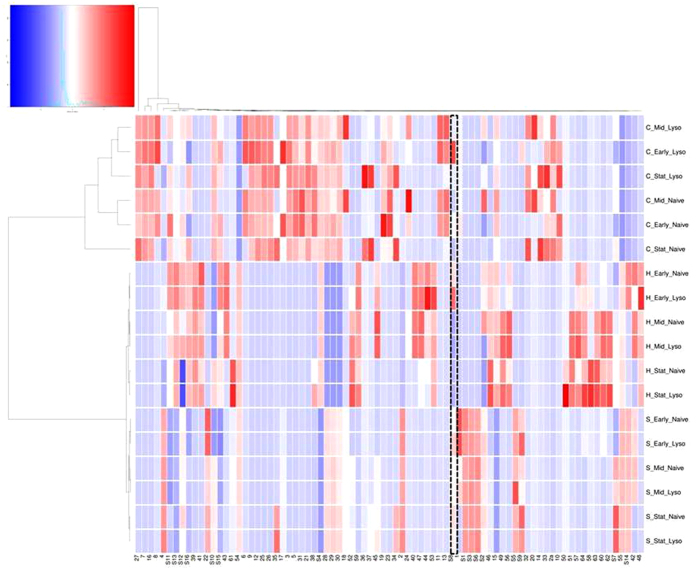
Heatmap generated by metabolic levels of 81 metabolites using HCA and DM Culture conditions and presence or absence of phage can be found alongside each profile (H = 8-hydroxyquinoline, C = chloroxylenol). Each individual tile represents a metabolite. The colour of a given tile denotes higher or lower intensity of the metabolite. The colour scale key is: dark blue: lowest levels; white: mid-point; dark red: highest level. The gradient between these colours represents variation in the levels of the metabolite across the colour scale (putative ID’s can be found in SI Table 3). Pimelic acid is highlighted across all profiles with a hatched box.

In the absence of antibiotics, the metabolite profile shows less discrimination between the lysogen and host at the 3 stages of growth by PLS-DA (Fig. 4A). Changes in individual metabolite abundances were measured as before (Figs 4 and 6), and >100 were deemed possible biologically relevant metabolites. From the confirmed compounds, a total of 16 metabolites (SI Table 3) were shown to discriminate between MC1061 and the ϕ24_B_ lysogen.

We further analysed these data using Hierarchical cluster analysis (HCA) and Euclidean dissimilarity matrix (DM) to create a heatmap that discriminates between 81 metabolites across all tests in this study (Fig. 6). The unsupervised heatmap shows that the metabolic profiles have separated by condition.

Figure 6 illustrates differences between the metabolic profiles of the naïve MC1061 and ϕ24_B_ lysogen when comparing both test antimicrobials and the standard culture conditions. Firstly there is the greatest dissimilarity when the naïve host or lysogen has been treated with a sub-inhibitory concentration of chloroxylenol. Within this grouping the naïve host shows the greatest difference in profile at stationary phase for the treatment group. The chloroxylenol group is further stratified by whether the phage is present or absent. Presence of the phage offers the most dissimilar metabolic profile under this antimicrobial challenge. Treatment with 8-hydroxyquinoline has less impact on the metabolic profiles, yet the antimicrobial tolerance is still marked. The difference is also less marked as the profiles are stratified by growth phase rather than presence or absence of phage. Importantly, in Fig. 6 differences between the 81 metabolites in the naïve host and lysogen without challenging with an antimicrobial are still apparent.

## Discussion

The accessory genome of bacteria promoted through horizontal gene transfer is important in understanding how mobile genetic elements aid selection in the environment. Metagenomics of DNA viruses in environmental and clinical samples has revealed a wide range of antimicrobial resistance genes (ARGs)^46^,^47^,^48^. Enault *et al*.^49^ demonstrate that caution is needed as ARGs are over-estimated and therefore rarely found in phage genomes and that this over-estimation was further supported by functionality^49^. We here propose a different mechanism promoted by Stx-phage ϕ24_B_, through infection and subversion of the cell physiology, promoting tolerance to sub-inhibitory concentrations of antimicrobials 8-hydroxyquinoline and chloroxylenol. Importantly, we show here that this tolerance is to antimicrobials commonly used globally in the farming industry. Chloroxylenol is widely used in detergent based products l, for the direct treatment of livestock e.g. bovine teat treatment^50^. Similarly 8-hydroxyquinoline has a broad antimicrobial activity with agricultural use due to its potency against insects, fungi, and bacteria^51^.

DeSmet *et al*., (2016) illustrated metabolomic differences during phage infection of *P. aeruginosa*^52^, whereas this is the first reported use of a metabolic profiling approach to characterise the impact of temperate phage infection on the physiology of the bacteria under antimicrobial pressure. The impact of prophage should not be underestimated as basis for metabolic variation and selection for the bacterial host by heightening or dampening cellular response to stress. With the altered metabolic profile of the ϕ24_B_ lysogen and increased levels of biotin concentration and fatty acid intensities it leads to the hypothesis that altered growth and lipids may play a role in altering the cell surface that promotes antimicrobial exclusion.

The metabolite pimelic acid is a precursor for the majority of the carbon atoms of biotin^53^. Biotin plays a crucial role in cell metabolism via carboxylation and decarboxylation reactions. Beyond its function as a cofactor for carboxylases, biotin also plays a role in gene regulation in mammals^54^. Unfortunately the mechanism of its action in *E. coli* is relatively unknown. However it has been shown that the *E. coli* BioC–BioH pathway uses a methylation and demethylation strategy to complete the necessary pimeloyl moiety^53^. This methylation approach disguises the biotin synthetic intermediates such that they become substrates for the fatty acid synthetic pathway, and once the pimeloyl moiety is complete it is demethylated^53^. We show here that the ϕ24_B_ prophage has a significant upregulatory effect on biotin that links to other physiological pathways including fatty acid synthesis^53^. Differences in pimelic acid intensities between lysogen and naïve host were greatest during early growth (Figs 1A, 2B, 3A and 5A), which correlates with the differences observed in growth rates during the first 2.5 hours of culture (Fig. 1). This is associated with a ~3 fold increase in the level of biotin present per cell at mid-exponential growth phase (Fig. 5B3), which correlates to metabolite profiling for pimelic acid. This is the first time a phage has been shown to drive the biotin pathway.

The Biolog data confirmed significant differences in rates of respiration between the naïve host and lysogen under different nutrient and chemical challenges. Interestingly the ϕ24_B_ lysogens acquired the ability to respire using Uridine-2-Phosphate where the AURC is illustrated in Fig. 2A and SI Figure 1. U-2-P is involved in cellular metabolism (including biotin metabolism), nucleotide metabolism, pyrimidine metabolism and pyrimidine catabolism^55^. Phages have been shown to subvert purine and pyrimidine synthesis to aid viral construction and proliferation^52^,^56^. Lysogen mediated differences encoded by ϕ24_B_ also supports these numerous studies as metabolomics profiling identifies increased pyrimidine catabolism as 5-methyluridine intensity decreases in the lysogen sample. It has also been previously shown through metagenomic analysis that well adapted phages of *Pseudomonas aeruginosa* in the lung carry genes that are involved with purine, pyrimidine and different phosphate utilisation^57^. This is further supported by Chevallereau, *et al*.^58^ who show marked changes in RNA metabolism during bacteriophage infection of *P. aeruginosa*^58^. Importantly, not only does ϕ24_B_ lysogen show increased pyrimidine utilisation, it also shows that phages can expand the group of phosphates *E. coli* can use for cell respiration and growth, in this instance U-2-P.

Conversely to addition of function, integration of ϕ24_B_ into the MC1061 chromosome confers an inability to respire using β-D- allose. The lysogen used in this study has ϕ24_B_ inserted into the primary integration site on the *E. coli* genome ~250 bp downstream of *intS*^45^. In *E. coli* there are 3 genes, alsB, alsA, and alsC, that are linked to the utilisation of D-allose^59^, but are disparate (~700 Kbp) from any of the 6 integration sites reported by Fogg *et al*.^43^,^45^. This is significant as it illustrates that integration can yield off target epigenetic effects. This study illustrates that the lysogen associated changes in fatty acid synthesis may exclude D-allose being transported into the bacterial cell, although the mechanism of this restriction is unknown.

Previous research showed infection with λ increased cell growth by the lysogen under cell starvation/supplementation of glucose in a chemostat culture^60^,^61^,^62^. This increase in growth rate is also seen with ϕ24_B_ here. Interestingly we see a further increase with infection of a secondary, genetically identical phage. The double lysogen is an identical clone to that reported by Fogg *et al*.^43^, with phage integrating into the secondary integration site^45^. When monitoring growth the single lysogen confers a doubling time of 17 mins compared to 20 mins for the naïve MC1061. This is also combined with shorter lag phases in both the single and double lysogen. It has previously been shown in many bacteria and yeast that augmenting a growing culture with biotin increases cell growth rates^63^,^64^,^65^,^66^,^67^.

When stressed with chloroxylenol, a demonstrated increase in lipid biosynthesis occurs in the lysogen presumably through subversion of the biotin pathway. This is supported through identification of higher intensity levels of hexadecanoic acid in the metabolite data. Hexadecanoic acid is involved in the biosynthesis of lipid A, a core outer cell membrane structure^68^,^69^,^70^. Changes in hexadecanoic acid in the lipid A structure of *E. coli* have been previously shown to be associated to mutations in the firA gene^69^,^71^. The firA gene is essential for growth and outer membrane synthesis^72^, and is essential for rifampicin resistance associated with certain mutations in the β subunit of the RNA polymerase^70^. This resistance and increase in hexadecanoic acid associated to the firA gene, shows that manipulation of this specific fatty acid likely improves antibiotic resistance. It is noteworthy that altering cell wall properties can broadly improve drug resistance^73^, and the biotin pathway is intrinsically linked to cell wall synthesis and growth^53^,^74^.

The lysogen showed increased tolerance to 8-hydroxyquinoline and chloroxylenol using the Biolog phenotypic array and sub-inhibitory antimicrobial tests. An untargeted metabolomics approach demonstrated that phage conversion offers the bacterial host different metabolic profiles to tolerate the two antimicrobials tested. The tolerance observed also suggests core functional changes allowing the cell to resist two disparate antimicrobials. This may suggest that these lysogen associated metabolic differences would likely infer tolerance to other environmental challenges and selective pressures.

Firstly, we present a metabolic difference in growth under standard culture conditions between the naïve MC1061 and the ϕ24_B_ lysogen. From 81 metabolites, 16 were discriminatory between the lysogen and MC1061 (SI Table 3). Pimelic acid is present and constitutively raised after infection by ϕ24_B_. We also showed here a difference between metabolites at the 3 key stages of growth. These again differ between the lysogen and MC1061 (Fig. 6).

Under treatment with chloroxylenol in early growth, increased intensity of hexadecanoic acid was identified, particularly in the lysogen. The metabolite sphinganine was observed in our data, sphinganine plays an essential part in the sphingolipid synthesis pathway^75^. In both the lysogen and naïve host there is evidence of higher intensities of a sphinganine under chloroxylenol treatment and at stationary growth in standard conditions (Fig. 6). In *Shigella* species, a pathway associated with mammalian sphingolipid based rafts has been linked to improved binding and mammalian host cell entry^76^.

Metabolic differences between the lysogen and naïve bacteria are the most disparate when under challenge of a sub-inhibitory concentration of chloroxylenol, illustrated in the PLS-DA plots (Fig. 4) and heatmap (Fig. 6). Chloroxylenol is a bactericidal agent and a halophenol that targets microbial membranes^73^ with a broad activity as an antimicrobial^77^.

We also demonstrate an increased tolerance by the lysogen to antimicrobial 8-hydroxyquinoline. Interestingly, compared to stress under chloroxylenol, the 8-hydroxyquinoline tested metabolite profile changes less significantly from standard conditions. Furthermore when treated with 8-hydroxyquinoline, the metabolite profile is less pronounced in the lysogen when compared to the chloroxylenol test. 8-hydroxyquinoline is a lipophilic metal-chelator with intracellular targets^78^. It inhibits growth by chelating metal ions, e.g. Zn^2+^ on RNA polymerase^79^,^80^. The changes in the intensity of lipids present at the cell surface, that we previously suggested affect uptake of D-allose, are similarly likely to inhibit levels of these 2 antimicrobials entering the cell.

When testing cellular stress it is imperative to find markers of inhibition detailed in the metabolite data. The metabolomic profiles identified 2 discriminatory metabolites that are associated with cellular stress: FAPy-Adenine^47^ and ophthalmic acid^81^,^82^. Our data showed that FAPy-Adenine was only present when cells were challenged by the antimicrobials 8-hydroxyquinoline and chloroxylenol. Interestingly the intensity levels of FAPy-adenine differ greatly depending on the antimicrobial used and also presence or absence of integrated ϕ24_B_ (Fig. 5). In the presence of chloroxylenol the lysogen demonstrates lower intensities of the stress marker FAPy-adenine, 0.56 and 0.37 fold less in early and mid-exponential growth phase respectively (Figs 2a,b and 5). It also shows higher intensity of pimelic acid compared to MC1061. This strengthens the hypothesis of a biotin related lipid increase or change at the cell surface lowering levels of the drug reaching its intracellular target.

When challenging the culture with 8-hydroxyquinoline, FAPy-adenine intensity increases rapidly, even more so than the naïve host (Fig. 5). Again there is an increase in pimelic acid intensity that is ubiquitous to the metabolic profiles in the presence of an integrated ϕ24_B_. However hexadecanoic acid was undetectable within the cell, which may be associated with the lipophilic nature of the drug. The stress response occurs directly after treating with 8-hydroxyquinoline and likely promotes some cell death. Extracellular lipids released through cell lysis binds the drug, forming a matrix. This therefore would reduce the concentration of the available drug present allowing the bacterial culture to grow.

The second stress marker ophthalmic acid is an analogue of glutathione and a reported marker of oxidative stress^81^,^82^. Ophthalmic acid intensity mirrored the stationary phase levels of FAPy-adenine, across all tests, however it was also present in standard culture conditions in both the early and mid-exponential growth phase cultures. This observation from our metabolomic analysis implies higher oxidative stress in the lysogen at stationary growth, as the data is supported by Desnues *et al*.^82^. The oxidative stress also correlates with the reduction in growth rate and a reduction in the intensity of pimelic acid.

This study has established that Stx-phage ϕ24_B_ provides a ‘jump start’ in early respiration and increased bacterial growth rates. These phage-mediated alterations in bacterial host metabolic profile may offer positive selection for the lysogen. Subversion of the biotin pathway is core to the changes mediated by ϕ24_B_ as it links to the bacterium becoming able to tolerate chloroxylenol and 8-hydroxyquinoline during early and mid-exponential growth phase. These tolerances are important as both antimicrobials are used globally in livestock farming. Importantly, metabolic shift and subversion offers 2 mechanisms of controlling this antimicrobial tolerance through increased biotin and fatty acid synthesis. With treatment and tolerance to choloroxylenol, alteration in levels of Lipid A, and presumably other lipids enables exclusion of the drug from entry. Secondly 8-hydroxyquinoline treatment drives early cellular stress, cell death and lysis which increases extracellular lipids that bind free drug, allowing the community to continue to grow.

The mechanism for this is unclear, yet this is importantly linked to subversion of the bacterial cell because no phage encoded metabolites are present. We therefore propose that temperate phages may not carry ARGs but play a larger role interfering with metabolic regulation that alters bacterial sensitivity to antimicrobials.

## Materials and Methods

### Bacterial strains and growth conditions - Buffer and Agar

All bacterial strains were grown in Lysogeny Broth + 0.01 M CaCl_2_ (LB). Growth of the MC1061(ϕ24_B_::Kan), a lysogen of the bacteriophage ϕ24_B_::Kan and growth of the MC1061(ϕ24_B_::Cat), a lysogen of the bacteriophage ϕ24_B_::Cat was supplemented with 50 μg.ml^−1^ kanamycin (kan) and Chloramphenicol (cat) respectively. Bottom agar plates for plaque assay included LB broth including 7% (w/v) grade 1 agar. Soft top agar was contained LB broth plus 0.4% (w/v) grade 1 agar. Unless otherwise stated culture conditions were at 37 °C, and broth cultures were shaken at 200 rpm.

### Growth curve of single and double lysogens

A single colony of either naïve MC1061, single or double lysogen was cultured overnight for 18 h (200 rpm). LB with 0.01 M CaCl_2_ (100 ml) was inoculated with 1% (v/v) of the overnight culture. Samples were taken over a 7 hour period, subject to serial, ten-fold dilutions and spread plated on LB agar plates.

### Bacterial phenotypic microarray - Biolog

The Biolog assay utilises a redox dye where a tetrazolium violet salt acts as an electron receptor from the tricarboxylic cycle and reduction to NADH. The transfer alters the clear salt to a purple formazan dye that is inexplicably linked to the cellular activity, specifically cell respiration. An inoculum was taken from an 18 h streaked plate of either MC1061 or ϕ24_B_ lysogen, raised through 2 rounds of passage from single colony amplification from cryo-stock. A single colony was added to fluid IF-0 (containing 50 μM leucine due to MC1061’s auxotrophy), to a transmittance of 42% T on a Biolog turbidometer in a 20 mm diameter tube as per manufacturer’s instructions and used to inoculate Biolog Phenotypic Microarray plates.

The panel plates used for this study included Biolog plates PM 1–20, which include a range of both metabolic and toxicological additives (see SI). Further details of the components associated with these PM plates can be found at http://www.biolog.com/pmMicrobialCells.html. The Biolog PM plates were grown at 37 °C and monitored using the Omnilog plate reader at 30 min intervals over 47 hours.

### Sub-inhibitory concentration (SIC) assay

An inoculum with a transmittance of 42 % T was created in an identical manner to that performed for the Biolog assay for both MC1061 or the lysogen. An identical volume of inoculum was added to an equal volume of antimicrobial (double concentrate) diluted in LB broth. Readings were taken at 0 and 18 hours; plates were incubated at 37 °C.

### SIC assay – Liquid chromatography mass spectrometry (LCMS) analysis comparing metabolic compounds from Naïve host and Lysogens

Replicated in triplicate therefore n = 9 bacterial cultures (10 ml) were grown as previously described under standard growth conditions and challenged with antimicrobials, the cells were harvested at early, mid, and late log phase. The cells were harvested by centrifugation (5,000 rpm for 5 min) and the pellet washed (×3) in ice cold 1 × PBS prior to lyophilisation. Lyophilised samples (×3) were pooled, normalised for weight/vol (normalised to 1 mg.ml) with methanol and 0.1% formic acid, this was vortexed and then sonicated (Bandelin Sonorex, Sonicator) for 1 hour and centrifuged (5 k rpm for 5 mins). The supernatant was recovered and filtered through 0.22 μm pore-sized, nylon filter and injected into the Q-Exactive LC-MS (Thermo-Fisher) after separation on a Phenomenex Gemini column (110 A, 150 × 2 mm, 5 μm, flow 0.2 ml.min). LCMS mobile phase parameters were: 0–6 mins at 20% ACN, 8 mins 60% ACN, 12 mins 95% ACN, 17 mins 95% ACN, 17.1 −23 mins 5% CAN. MS conditions were: full MS mode, resolution 70, 000, AGC target 1 × 10^6^, maximum IT 200 ms, scan range 150–2000, column temperature 35 °C. Progenesis QI software was used for raw data analysis; this software provided alignment, peak picking, pairwise statistical analysis and putative metabolite ID based on accurate mass. Metabolites were confirmed by analysing pure standards and MSMS fragmentation analysis run under identical analytical conditions.

### Biotin quantification assay

Inoculums were prepared in the same manner as for the SIC and Biolog assays. Optical density values were taken at 0 and 18 h, incubation at 37 °C. The cultures were diluted to the lowest OD_600_ reading to normalise cell number between naïve MC1061 and lysogen. Dilutions of both naïve MC1061 and lysogen were made in LB (1:100 and 1:1000). Cells were harvested by centrifugation (5,000 rpm for 5 mins). The biotin assay was completed using the Bio Vision® (Cambridge, UK) Biotin Quantitation Kit (Colorimetric) according to the manufacturer’s protocol. In brief, the supernatant was discarded and the pellet re-suspended in 10 μl PBS and heated to 100 °C for 3 min and then immediately placed on ice. Diluted naïve and lysogen cells (10 μl) were added to individual aliquots of Biotin Assay buffer (20 μl) and 300 μl of biotin reaction mix, pre-prepared as described in the Biotin Quantitation Kit protocol (version 7.6), was added to the buffer and cells. The mix was incubated at 21 °C for 15 min. Each sample mix (150 μl) was then pipetted into a microtitre plate and read at 500 nm. A standard curve was prepared as per manufacturer’s instructions.

### Statistical analysis

To determine statistically significant difference between growth rates of the single and double ϕ24_B_ lysogen compared to MC1061, paired-sample T-tests in the statistical package SPSS was used. The two tailed p values are given at 95% confidence limits. The statistically significant difference in SIC between lysogen and MC1061 was calculated using an independent t-test, using the SPSS platform (>95% confidence limits). The Biolog area under the respiration curve (AURC) for respiration values were calculated using a trapezoid algorithm. Statistical significance of area under the respiration curve (AURC) and comparison at a specific time point during mid exponential growth phase was achieved by determining normal Gaussian distribution by parametric analysis and statistical significance identified using an un-paired *t* test (>95% confidence limits). Metabolomic analysis was carried out primarily by Progenesis QI software, this software provided alignment, peak picking, pairwise statistical analysis and putative metabolite ID based on accurate mass. Further multivariate analysis was performed using SIMCA-P. ID’s were obtained through the QI plugin ’Progenesis metascope’ and filtered through a range of databases using sdf files (ECMDB, HMDB, small molecules drugs, Biomolecules, analgesics mix, Lipid MBD, Basic lipids, and Yeast DB). A paired sample t test was used to determine statistically significant differences between intensities of metabolites identified during metabolomic analyses (>95% confidence limits).

## Additional Information

**How to cite this article**: Holt, G. S. *et al*. Shigatoxin encoding Bacteriophage ϕ24_B_ modulates bacterial metabolism to raise antimicrobial tolerance. *Sci. Rep.* **7**, 40424; doi: 10.1038/srep40424 (2017).

**Publisher's note:** Springer Nature remains neutral with regard to jurisdictional claims in published maps and institutional affiliations.

## Supplementary Material

Supplementary Information

## References

[b1] Allison H. E. Stx-phages: drivers and mediators of the evolution of STEC and STEC-like pathogens. Future Microbiol. 2, 165–74 (2007). 17661653 10.2217/17460913.2.2.165

[b2] Muniesa M., Hammerl J. A., Hertwig S., Appel B. & Brussow H. Shiga Toxin-Producing Escherichia coli O104:H4: a New Challenge for Microbiology. Applied and environmental microbiology 78, 4065–73 (2012). 22504816 10.1128/AEM.00217-12 PMC3370534

[b3] Kawano K., Okada M., Haga T., Maeda K. & Goto Y. Relationship between pathogenicity for humans and stx genotype in Shiga toxin-producing Escherichia coli serotype O157. Eur. J. Clin. Microbiol. 27, 227–32 (2008). 10.1007/s10096-007-0420-3 18071766

[b4] O’Brien A. D. & Kaper J. B. Escherichia coli O157:H7 And Other Shiga Toxin-Producing E. coli Strains American Society for Microbiology Press, Washington, D.C. (O’Brien A. D. & Kaper J. B., 1998).

[b5] Johansen B. K., Wasteson Y., Granum P. E. & Brynestad S. Mosaic structure of Shiga-toxin-2-encoding phages isolated from Escherichia coli O157: H7 indicates frequent gene exchange between lambdoid phage genomes. Microbiol-Sgm. 147, 1929–36 (2001). 10.1099/00221287-147-7-1929 11429469

[b6] Jacewicz M. S. . Responses of human intestinal microvascular endothelial cells to shiga toxins 1 and 2 and pathogenesis of hemorrhagic colitis. Infection and immunity 67, 1439–44 (1999). 10024592 10.1128/iai.67.3.1439-1444.1999 PMC96478

[b7] Echols H. & Green L. Establishment and maintenance of repression by bacteriophage lambda: the role of the cI, cII, and c3 proteins. Proceedings of the National Academy of Sciences of the United States of America 68, 2190–4 (1971). 4943791 10.1073/pnas.68.9.2190 PMC389382

[b8] Takeda Y., Matsubara K. & Ogata K. Regulation of early gene expression in bacteriophage lambda: effect of tof mutation on strand-specific transcriptions. Virology 65, 374–84 (1975). 1129947 10.1016/0042-6822(75)90043-4

[b9] Reichardt L. F. Control of bacteriophage lambda repressor synthesis: regulation of the maintenance pathway of the cro and cI products. J. Mol. Biol. 93, 289–309 (1975). 1152054 10.1016/0022-2836(75)90133-3

[b10] Stern A. & Sorek R. The phage-host arms race: shaping the evolution of microbes. BioEssays: news and reviews in molecular, cellular and developmental biology 33, 43–51 (2011). 10.1002/bies.201000071 PMC3274958 20979102

[b11] Smith D. L. . Multilocus characterization scheme for shiga toxin-encoding bacteriophages. Applied and environmental microbiology 73, 8032–40 (2007). 17951439 10.1128/AEM.01278-07 PMC2168134

[b12] Bonanno L., Petit M. A., Loukiadis E., Michel V. & Auvray F. Heterogeneity in Induction Level, Infection Ability, and Morphology of Shiga Toxin-Encoding Phages (Stx Phages) from Dairy and Human Shiga Toxin-Producing Escherichia coli O26:H11 Isolates. Applied and environmental microbiology 82, 2177–86 (2016). 26826235 10.1128/AEM.03463-15 PMC4807521

[b13] Smith D. L. . Comparative genomics of Shiga toxin encoding bacteriophages. BMC genomics 13, 311 (2012). 22799768 10.1186/1471-2164-13-311 PMC3430580

[b14] Coren J. S., Pierce J. C. & Sternberg N. Headful Packaging Revisited - the Packaging of More Than One DNA Molecule into a Bacteriophage-P1 Head. Journal of Molecular Biology 249, 176–84 (1995). 7776370 10.1006/jmbi.1995.0287

[b15] Gottesma M., Gottesma M., Gottesma S. & Gellert M. Characterization of Bacteriophage-Lambda Reverse as an Escherichia-Coli Phage Carrying a Unique Set of Host-Derived Recombination Functions. Journal of Molecular Biology 88, 471-& (1974). 10.1016/0022-2836(74)90496-3 4616090

[b16] Wang X. . Cryptic prophages help bacteria cope with adverse environments. Nature communications 1, 147 (2010). 10.1038/ncomms1146 PMC3105296 21266997

[b17] Hayashi T. . Complete genome sequence of enterohemorrhagic Escherichia coli O157:H7 and genomic comparison with a laboratory strain K-12. DNA research: an international journal for rapid publication of reports on genes and genomes 8, 11–22 (2001). 11258796 10.1093/dnares/8.1.11

[b18] Allison H. E. . Immunity profiles of wild-type and recombinant shiga-like toxin-encoding bacteriophages and characterization of novel double lysogens. Infection and immunity 71, 3409–18 (2003). 12761125 10.1128/IAI.71.6.3409-3418.2003 PMC155745

[b19] Fogg P. C. M., Rigden D. J., Saunders J. R., McCarthy A. J. & Allison H. E. Characterization of the relationship between integrase, excisionase and antirepressor activities associated with a superinfecting Shiga toxin encoding bacteriophage. Nucleic Acids Research 39, 2116–29 (2011). 21062824 10.1093/nar/gkq923 PMC3064807

[b20] Fogg P. C., Allison H. E., Saunders J. R. & McCarthy A. J. Bacteriophage lambda: a paradigm revisited. Journal of virology 84, 6876–9 (2010). 20375161 10.1128/JVI.02177-09 PMC2903274

[b21] Smith D. L. . Short-tailed Stx phages exploit the conserved YaeT protein to disseminate Shiga Toxin genes among enterobacteria. J. Bacteriol. 189, 7223–33 (2007). 17693515 10.1128/JB.00824-07 PMC2168440

[b22] Johannessen G. S. . Survival of a Shiga toxin-encoding bacteriophage in a compost model. FEMS Microbiol Lett. 245, 369–75 (2005). 15837394 10.1016/j.femsle.2005.03.031

[b23] Nyambe S., Burgess C., Whyte P. & Bolton D. Survival studies of a temperate and lytic bacteriophage in bovine faeces and slurry. J. Appl. Microbiol. (2016). 10.1111/jam.13217 27371115

[b24] Willshaw G. A., Smith H. R., Scotland S. M. & Rowe B. Cloning of genes determining the production of vero cytotoxin by Escherichia coli. Journal of general microbiology 131, 3047–53 (1985). 3005476 10.1099/00221287-131-11-3047

[b25] Sakaguchi Y. . The genome sequence of Clostridium botulinum type C neurotoxin-converting phage and the molecular mechanisms of unstable lysogeny. Proceedings of the National Academy of Sciences of the United States of America 102, 17472–7 (2005). 16287978 10.1073/pnas.0505503102 PMC1283531

[b26] Davis B. M., Moyer K. E., Boyd E. F. & Waldor M. K. CTX prophages in classical biotype Vibrio cholerae: Functional phage genes but dysfunctional phage genomes. J. Bacteriol. 182, 6992–8 (2000). 11092860 10.1128/jb.182.24.6992-6998.2000 PMC94825

[b27] Unkmeir A. & Schmidt H. Structural analysis of phage-borne stx genes and their flanking sequences in Shiga toxin-producing Escherichia coli and Shigella dysenteriae type 1 strains. Infection and immunity 68, 4856–64 (2000). 10948097 10.1128/iai.68.9.4856-4864.2000 PMC101682

[b28] Livny J. & Friedman D. I., Characterizing spontaneous induction of Stx encoding phages using a selectable reporter system. Mol. Microbiol. 51, 1691–704 (2004). 15009895 10.1111/j.1365-2958.2003.03934.x

[b29] Colon M., Chakraborty D., Pevzner Y. & Koudelka G. Mechanisms that Determine the Differential Stability of Stx+ and Stx− Lysogens. Toxins 8, 96 (2016). 27043626 10.3390/toxins8040096 PMC4848623

[b30] McGrath S., Seegers J. F., Fitzgerald G. F. & van Sinderen D. Molecular characterization of a phage-encoded resistance system in Lactococcus lactis. Applied and environmental microbiology. 65, 1891–9 (1999). 10223975 10.1128/aem.65.5.1891-1899.1999 PMC91272

[b31] Colomer-Lluch M., Jofre J. & Muniesa M. Antibiotic Resistance Genes in the Bacteriophage DNA Fraction of Environmental Samples. Plos One. 6 (2011). 10.1371/journal.pone.0017549 PMC3048399 21390233

[b32] Veses-Garcia M. . Transcriptomic analysis of Shiga-toxigenic bacteriophage carriage reveals a profound regulatory effect on acid resistance in Escherichia coli. Applied and environmental microbiology 81, 8118–25 (2015). 26386055 10.1128/AEM.02034-15 PMC4651098

[b33] Su L. K., Lu C. P., Wang Y., Cao D. M., Sun J. H. & Yan Y. X. Lysogenic infection of a Shiga toxin 2-converting bacteriophage changes host gene expression, enhances host acid resistance and motility. Molekuliarnaia biologiia 44, 60–73 (2010). 20198860

[b34] Vostrov A. A., Vostrukhina O. A., Svarchevsky A. N. & Rybchin V. N. Proteins responsible for lysogenic conversion caused by coliphages N15 and phi80 are highly homologous. J. Bacteriol. 178, 1484–6 (1996). 8631731 10.1128/jb.178.5.1484-1486.1996 PMC177828

[b35] Reeve J. N. & Shaw J. E. Lambda encodes an outer membrane protein: the lom gene. Molecular & general genetics: MGG 172, 243–8 (1979). 45607 10.1007/BF00271723

[b36] Barondess J. J. & Beckwith J. A bacterial virulence determinant encoded by lysogenic coliphage lambda. Nature 346, 871–4 (1990). 2144037 10.1038/346871a0

[b37] Newland J. W., Strockbine N. A., Miller S. F., O’Brien A. D. & Holmes R. K. Cloning of Shiga-like toxin structural genes from a toxin converting phage of Escherichia coli. Science 230, 179–81 (1985). 2994228 10.1126/science.2994228

[b38] Sako T. . Cloning and expression of the staphylokinase gene of Staphylococcus aureus in Escherichia coli. Molecular & general genetics: MGG 190, 271–7 (1983). 6224069 10.1007/BF00330650

[b39] Nejman-Falenczyk B. . A small, microRNA-size, ribonucleic acid regulating gene expression and development of Shiga toxin-converting bacteriophage Phi24 Beta. Scientific reports 5, 10080, doi: 10.1038/srep10080 (2015). 25962117 PMC4426718

[b40] Tree J. J., Granneman S., McAteer S. P., Tollervey D. & Gally D. L. Identification of Bacteriophage-Encoded Anti-sRNAs in Pathogenic Escherichia coli. Mol. Cell 55, 199–213 (2014). 24910100 10.1016/j.molcel.2014.05.006 PMC4104026

[b41] Bochner B. R., Gadzinski P. & Panomitros E. Phenotype MicroArrays for high-throughput phenotypic testing and assay of gene function. Genome Res. 11, 1246–55 (2001). 11435407 10.1101/gr.186501 PMC311101

[b42] Khatri B. . High Throughput Phenotypic Analysis of Mycobacterium tuberculosis and Mycobacterium bovis Strains’ Metabolism Using Biolog Phenotype Microarrays. Plos One 8 (2013). 10.1371/journal.pone.0052673 PMC3542357 23326347

[b43] Fogg P. C. . Identification of multiple integration sites for Stx-phage Phi24B in the Escherichia coli genome, description of a novel integrase and evidence for a functional anti-repressor. Microbiology. 153, 4098–110 (2007). 18048923 10.1099/mic.0.2007/011205-0

[b44] Matsushiro A., Sato K., Miyamoto H., Yamamura T. & Honda T. Induction of prophages of enterohemorrhagic Escherichia coli O157: H7 with norfloxacin. J. Bacteriol. 181, 2257–60 (1999). 10094706 10.1128/jb.181.7.2257-2260.1999 PMC93641

[b45] Graziewicz M. A. . Fapyadenine is a moderately efficient chain terminator for prokaryotic DNA polymerases. Free Radical Bio Med. 28, 75–83 (2000). 10656293 10.1016/s0891-5849(99)00208-7

[b46] Parsley L. C. . Identification of diverse antimicrobial resistance determinants carried on bacterial, plasmid, or viral metagenomes from an activated sludge microbial assemblage. Applied and environmental microbiology 76, 3753–7 (2010). 20382816 10.1128/AEM.03080-09 PMC2876469

[b47] Marti E., Variatza E. & Balcazar J. L. Bacteriophages as a reservoir of extended-spectrum beta-lactamase and fluoroquinolone resistance genes in the environment. Clin. Microbiol. Infec. 20, O456–O9 (2014). 24552593 10.1111/1469-0691.12446

[b48] Colomer-Lluch M., Jofre J. & Muniesa M. Quinolone resistance genes (qnrA and qnrS) in bacteriophage particles from wastewater samples and the effect of inducing agents on packaged antibiotic resistance genes. J. Antimicrob. Chemother 69, 1265–74 (2014). 24458509 10.1093/jac/dkt528

[b49] Enault F. . Phages rarely encode antibiotic resistance genes: a cautionary tale for virome analyses. ISME J. 10.1038/ismej.2016.90 (2016). PMC5315482 27326545

[b50] Ascenzi J. Handbook of Disinfectants and Antiseptics (ed. Ascenzi J.) 265–291 (1996).

[b51] Al-Busafi S., Suliman F. & Al-Alawi Z. 8-Hydroxyquinoline and its Derivatives: Synthesis and Applications. Research and Reviews: Journal of Chemistry 3 (2014).

[b52] De Smet J. . High coverage metabolomics analysis reveals phage-specific alterations to Pseudomonas aeruginosa physiology during infection. ISME J. 10, 1823–35 (2016). 26882266 10.1038/ismej.2016.3 PMC5029163

[b53] Lin S., Hanson R. E. & Cronan J. E. Biotin synthesis begins by hijacking the fatty acid synthetic pathway. Nat. Chem. Biol. 6, 682–8 (2010). 20693992 10.1038/nchembio.420 PMC2925990

[b54] Dakshinamurti K. Biotin - a regulator of gene expression. J Nutr Biochem. 16, 419–23 (2005). 15992682 10.1016/j.jnutbio.2005.03.015

[b55] Kanehisa M. & Goto S. KEGG: Kyoto Encyclopedia of Genes and Genomes. Nucleic Acids Research 28, 27–30 (2000). 10592173 10.1093/nar/28.1.27 PMC102409

[b56] Friedman S. & Gots J. S. The purine and pyrimidine metabolism of normal and phage-infected Escherichia coli. J. Biol. Chem. 201, 125–35 (1953). 13044781

[b57] Tariq M. A. . A metagenomic approach to characterize temperate bacteriophage populations from Cystic Fibrosis and non-Cystic Fibrosis bronchiectasis patients. Frontiers in microbiology 6, 97 (2015). 25741327 10.3389/fmicb.2015.00097 PMC4332376

[b58] Chevallereau A. . Next-Generation “-omics” Approaches Reveal a Massive Alteration of Host RNA Metabolism during Bacteriophage Infection of Pseudomonas aeruginosa. PLoS genetics. 12, e1006134 (2016). 27380413 10.1371/journal.pgen.1006134 PMC4933390

[b59] Kim C. H., Song S. G. & Park C. The D-allose operon of Escherichia coli K-12. J. Bacteriol. 179, 7631–7 (1997). 9401019 10.1128/jb.179.24.7631-7637.1997 PMC179723

[b60] Edlin G., Lin L. & Kudrna R. Lambda Lysogens of Escherichia-Coli Reproduce More Rapidly Than Non-Lysogens. Nature 255, 735–7 (1975). 1094307 10.1038/255735a0

[b61] Lin L., Bitner R. & Edlin G. Increased Reproductive Fitness of Escherichia-Coli Lambda-Lysogens. Journal of virology 21, 554–9 (1977). 319255 10.1128/jvi.21.2.554-559.1977 PMC353857

[b62] Edlin G., Lin L. & Bitner R. Reproductive Fitness of P1, P2, and Mu-Lysogens of Escherichia-Coli. Journal of virology 21, 560–4 (1977). 319256 10.1128/jvi.21.2.560-564.1977 PMC353858

[b63] Snell E. E. & Mitchell H. K. Purine and pyrimidine bases as growth substances for lactic acid bacteria. Proceedings of the national academy of sciences 27, 1 (1940). 10.1073/pnas.27.1.1 PMC1078262 16588417

[b64] Underkofler L. A., Bantz A. C. & Peterson W. H. Growth Factors for Bacteria: XIV. Growth Requirements of Acetobacter suboxydans. J. Bacteriol. 45, 183–90 (1943). 16560624 10.1128/jb.45.2.183-190.1943 PMC373730

[b65] Williams R. J., Eakin R. E. & Snell E. E. The Relationship of Inositol, Thiamin, Biotin, Pantothenic Acid and Vitamin B6 to the Growth of Yeasts. Journal of the American Chemical Society 62, 1204–7 (1940).

[b66] Porter J. R. & Pelczar M. J. The Nutrition of Staphylococcus aureus: The Influence of Biotin, Bios II(B) and Vitamin H on the Growth of Several Strains. J. Bacteriol. 41, 173–92 (1941). 16560395 10.1128/jb.41.2.173-192.1941 PMC374689

[b67] Kögl F. & Tönnis B. Über das Bios-Problem. Darstellung von krystallisiertem Biotin aus Eigelb. 20. Mitteilung über pflanzliche Wachstumsstoffe. Hoppe-Seyler´ s Zeitschrift für physiologische Chemie 242, 43–73 (1936).

[b68] Hansen-Hagge V. L., Seydel U., Lindner B. & Zähringer U. Isolation and structural analysis of two lipid A precursors from a KDO deficient mutant of Salmonella typhimurium differing in their hexadecanoic acid content. Archives of microbiology 141, 353–8 (1985). 3893354 10.1007/BF00428849

[b69] Helander I. M., Lindner B., Seydel U. & Vaara M. Defective biosynthesis of the lipid A component of temperature-sensitive firA (omsA) mutant of Escherichia coli. Eur. J. Biochem. 212, 363–9 (1993). 8444173 10.1111/j.1432-1033.1993.tb17670.x

[b70] Lathe R. & Lecocq J. P. The firA gene, a locus involved in the expression of rifampicin resistance in Escherichia coli. II. Characterisation of bacterial proteins coded by lambdafirA transducing phages. Molecular & general genetics: MGG 154, 53–60 (1977). 19693 10.1007/BF00265576

[b71] Roy A. M. & Coleman J. Mutations in firA, encoding the second acyltransferase in lipopolysaccharide biosynthesis, affect multiple steps in lipopolysaccharide biosynthesis. J. Bacteriol. 176, 1639–46 (1994). 8132458 10.1128/jb.176.6.1639-1646.1994 PMC205249

[b72] Vuorio R. & Vaara M. Mutants Carrying Conditionally Lethal Mutations in Outer-Membrane Genes Omsa and Fira (Ssc) Are Phenotypically Similar, and Omsa Is Allelic to Fira. J. Bacteriol. 174, 7090–7 (1992). 1429432 10.1128/jb.174.22.7090-7097.1992 PMC207397

[b73] McDonnell G. & Russell A. D. Antiseptics and disinfectants: activity, action, and resistance. Clin. Microbiol Rev. 12, 147–79 (1999). 9880479 10.1128/cmr.12.1.147 PMC88911

[b74] Zhang Y. M. & Rock C. O. Membrane lipid homeostasis in bacteria. Nat. Rev. Microbiol. 6, 222–33 (2008). 18264115 10.1038/nrmicro1839

[b75] Futerman A. H. & Riezman H. The ins and outs of sphingolipid synthesis. Trends. Cell. Biol. 15, 312–8 (2005). 15953549 10.1016/j.tcb.2005.04.006

[b76] Lafont F., Van Nhieu G. T., Hanada K., Sansonetti P. & van der Goot F. G. Initial steps of Shigella infection depend on the cholesterol/sphingolipid raft‐mediated CD44–IpaB interaction. The EMBO Journal. 21, 4449–57 (2002). 12198147 10.1093/emboj/cdf457 PMC126195

[b77] Darouiche R. O. Method of coating medical devices with a combination of antiseptics and antiseptic coating therefor. Google Patents (2000).

[b78] Short B. R. D., Vargas M. A., Thomas J. C., O’Hanlon S. & Enright M. C. *In vitro* activity of a novel compound, the metal ion chelating agent AQ(+), against clinical isolates of Staphylococcus aureus. J. Antimicrob. Chemoth. 57, 104–9 (2006). 10.1093/jac/dki428 16319182

[b79] Collins J. J., Adler C. R., Fernandezpol J. A., Court D. & Johnson G. S. Transient Growth-Inhibition of Escherichia-Coli K-12 by Ion Chelators – *In vivo* Inhibition of Ribonucleic-Acid Synthesis. J. Bacteriol. 138, 923–32 (1979). 110773 10.1128/jb.138.3.923-932.1979 PMC218123

[b80] Fraser R. S. & Creanor J. The mechanism of inhibition of ribonucleic acid synthesis by 8-hydroxyquinoline and the antibiotic lomofungin. Biochem. J. 147, 401–10 (1975). 810137 10.1042/bj1470401 PMC1165465

[b81] Soga T. . Differential metabolomics reveals ophthalmic acid as an oxidative stress biomarker indicating hepatic glutathione consumption. J. Biol. Chem. 281, 16768–76 (2006). 16608839 10.1074/jbc.M601876200

[b82] Desnues B. . Differential oxidative damage and expression of stress defence regulons in culturable and non-culturable Escherichia coli cells. Embo Rep. 4, 400–4 (2003). 12671690 10.1038/sj.embor.embor799 PMC1319155

