# Supplementary material for: Shigatoxin encoding Bacteriophage ϕ24B modulates bacterial metabolism to raise antimicrobial tolerance
Source: Sci Rep. 2017 Jan 20;7:40424. doi: 10.1038/srep40424 (PMC5247750; doi:10.1038/srep40424)
Supplement: Supplementary Information [file srep40424-s1.pdf]

Shigatoxin encoding Bacteriophage ϕ24$_B$ modulates bacterial metabolism to raise antimicrobial tolerance

Holt, G. S.[1], Lodge, J.[1], McCarthy, A. J.[2], Graham, A.K.[1], Young, G[1]. Bridge, S. H.[1], Brown, A. K.[4], Veses-Garcia, M.[2], 3 Lanyon, C.V.[1], Sails, A.[3], Allison, H. E.[2*] and Smith, D. L.[1, 2*ǂ]

[1]Faculty of Health and Life Sciences, Northumbria University, Newcastle upon Tyne, UK

[2]Microbiology Research Group, Institute of Integrative Biology, University of Liverpool, Liverpool, UK

[3]Public Health England, Royal Victoria Hospital, Newcastle upon Tyne, UK

[4]School of Medicine, Pharmacy and Health, Durham University, Durham UK

[*]Co-senior authors

ǂ Corresponding author Darren.smith@northumbria.ac.uk

Supplementary Information (SI)

SI Table 1.

Cell Growth (single and double lysogen compared to uninfected host)			
Time (h)	Single or double lysogen	P value	Test type
1	$\Phi24_B$Kan	<0.001	Paired T-test (SPSS)
	$\Phi24_B$KanCat	<0.001	
1.5	$\Phi24_B$Kan	<0.001	
	$\Phi24_B$KanCat	0.016	
2.5	$\Phi24_B$Kan	<0.001	
	$\Phi24_B$KanCat	<0.001	
3	$\Phi24_B$Kan	0.014	
	$\Phi24_B$KanCat	<0.001	
4	$\Phi24_B$Kan	<0.001	
	$\Phi24_B$KanCat	<0.001	
5	$\Phi24_B$Kan	<0.001	
	$\Phi24_B$KanCat	<0.001	
6	$\Phi24_B$Kan	<0.001	
	$\Phi24_B$KanCat	<0.001	
7	$\Phi24_B$Kan	0.02	
	$\Phi24_B$KanCat	<0.001	
SIC			
Condition	Concentration (µmolar)	P value	Test type
8-Hydroxyquinoline	27.6	0.019	Independent samples T-test (SPSS)
	29.3	<0.001	
	31	<0.001	
	32.72	<0.001	
	34.4	<0.001	
	36.2	<0.001	
Chloroxylenol (4-chlor-3,5-dimethylphenol)	28.7	<0.001	
	35.1	<0.001	
	41.5	0.023	
Oxolinic acid	0.038	<0.001	
	0.057	<0.001	
	0.077	<0.001	
	0.096	<0.001	
	0.115	<0.001	
	0.134	<0.001	
FAPy-adenine			
Condition	Growth phase	P value	Test type
8-Hydroxyquinoline	Early	0.45	Paired T-test (SPSS)
	Mid	0.93	
	Stationary	<0.001	
Chloroxylenol (4-chlor-3,5-dimethylphenol)	Early	<0.001	
	Mid	0.002	
	Stationary	0.009	
Pimelic acid			
Condition	Growth phase	P value	Test type
8-Hydroxyquinoline	Early	0.02	Paired T-test (SPSS)
	Mid	N/A	
	Stationary	0.012	
Chloroxylenol (4-chlor-3,5-dimethylphenol)	Early	<0.001	
	Mid	0.07	
	Stationary	N/A	

SI Table 2. List of selected parameters where (with white background) the lysogen respired preferentially under these selected conditions compared to naïve MC1061. The compounds in the shaded area are those where the naïve MC1061 host respired preferentially compared to the lysogen.

Test	Altered use of nutrient source or targeting antimicrobial where a difference was seen between lysogen and naïve MC1061	Statistical Difference AUC	Statistical difference at mid-exponential growth phase (18h)
Uridine 2'-Monophosphate	P-Source, nucleotide, pyrimidine, uracil, Phosphate	P= 0.0094	P=0.0173
8-Hydroxyquinoline	Chelator lipophilic, RNA synthesis	p=<0.0001	p=<0.0001
Chloroxylenol	Fungicide	p=0.0032	p=0.0037
Cefoxitin	wall, cephalosporin second generation	p=0.015	p=0.0361
Puromycin	protein synthesis, 30S ribosomal subunit, premature chanin termination	p=0.0168	p=0.154
Niaproof	membrane, detergent, anionic	P=0.0192	P=0.0132
Geneticin (G418)	protein synthesis, aminoglycoside	p=0.02	p=0.0146
Cefamandole	wall, cephalosporin second generation	p=0.0239	p=0.1031
Amoxicillin	wall, lactam	p=0.057	p=0.0342
Cefmetazole	wall, cephalosporin second generation	p=0.08	p=0.0026
Methyltrioctylammonium chloride	membrane, detergent, cationic	p=0.08	p=0.777
Chlorhexidine	membrane, electron transport	p=0.12	p=0.14
Ceftriaxone	wall, cephalosporin third generation	p=0.14	p=0.0791
Phenylarsine oxide	tyrosine phosphatase inhibitor	p=0.17	p=0.2293
Penicillin G	wall, lactam	p=0.25	p=0.33
Cefuroxime	wall, cephalosporin second generation	P=0.27	P=0.0174
Moxalactam	wall, lactam	p=0.59	p=0.077
Cefazolin	wall, cephalosporin first generation	p=0.61	P=0.55
β-D-Allose	C-Source,carbohydrate,pentose	P=0.0097	P=0.0001
Ofloxacin	DNA unwinding, gyrase (GN), topoisomerase (GP), fluoroquinolone	p=0.0048	p=0.0008
Oxolinic acid	DNA unwinding, gyrase (GN), topoisomerase (GP), quinolone	P=0.0066	P=0.0274
6% Potassium Chloride	osmotic sensitivity, KCl	P=0.0631	P=0.073

Lomefloxacin	DNA unwinding, gyrase (GN), topoisomerase (GP), fluoroquinolone	p=0.09	p=0.09
5% Potassium Chloride	osmotic sensitivity, KCl	P=0.0939	P=0.565
4% NaCl	osmotic sensitivity, NaCl	p=0.1042	p=0.134
3% NaCl	osmotic sensitivity, NaCl	p=0.1188	p=0.0699
Sodium salicylate	anti-capsule, biofilm inhibition, mar inducer	p=0.1384	P=0.055
Ciprofloxacin	DNA unwinding, gyrase (GN), topoisomerase (GP), fluoroquinolone	p=0.255	P=0.37
2% Sodium Lactate	osmotic sensitivity, sodium lactate	p=0.2675	p=0.0656
5% NaCl	osmotic sensitivity, NaCl	p=0.44	p=0.2456
Sodium metasilicate	toxic anion	P=0.7	p=0.11

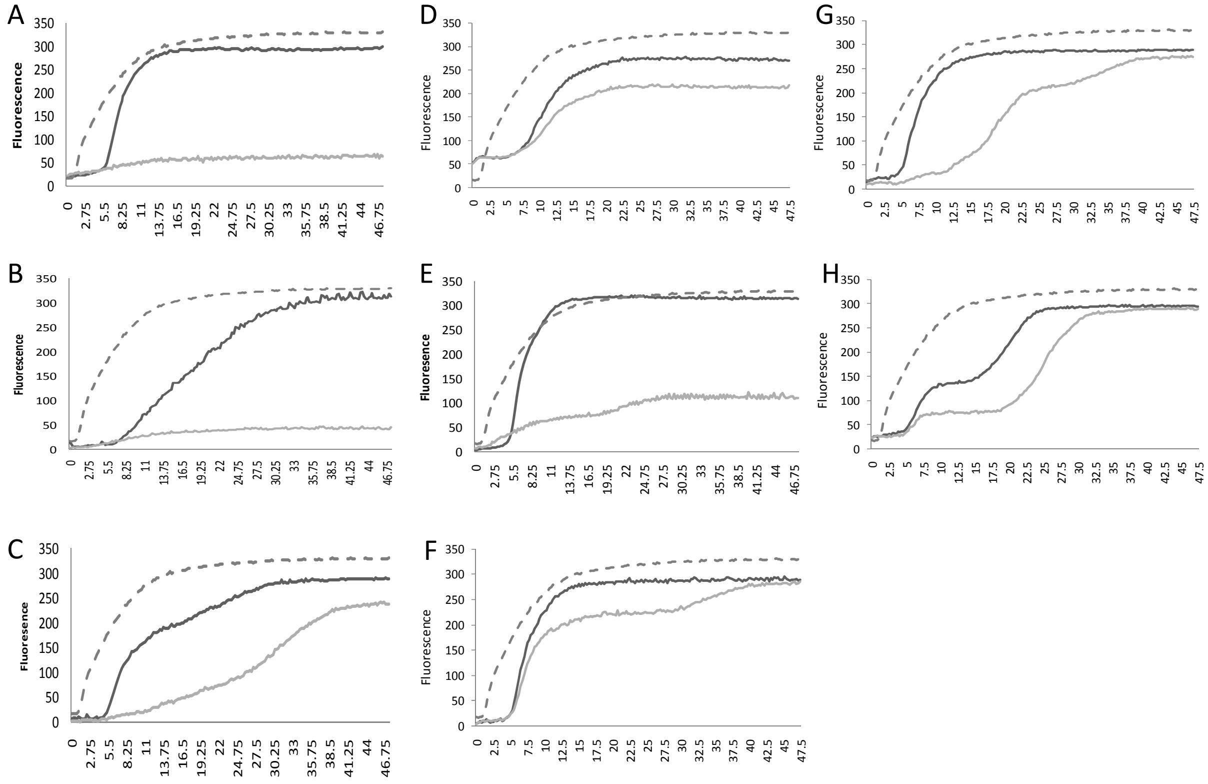

SI Figure 1 Respiration traces from raw Biolog data comparing naïve MC1061 respiration (light grey line) to lysogen (dark grey line), the hashed line represents (n=3)rates of respiration of both naïve MC1061under standard growth conditions in the absence of challenge. Panel **A** illustrates the lysogen's ability to now utilise a different phosphate source for respiration. **B-H** show respiration in the presence of other antimicrobials. Test compounds; **A** - Uridine 2-monophosphate; **B** - 8- Hydroxyquinoline; **C** – Chloroxylenol; **D** – Cefoxitin; **E** – Niaproof; **F** – Cefamandole; **G**-Amoxicillin; **H** – Cefmetazole. Statistically significant differences using area under the curve can be found in Table 2.

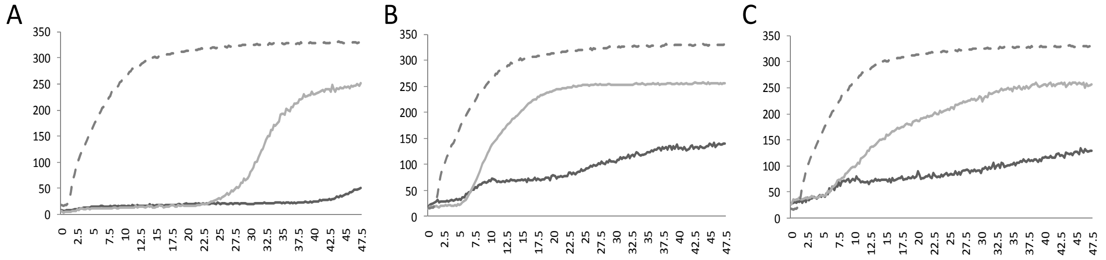

SI Figure 2 Respiration traces from raw Biolog data comparing naïve MC1061 respiration (light grey line) to lysogen (dark grey line), the hashed line represents (n=6) the combine respiration control data of both naïve MC1061 and Lysogen. Test compounds; A - b-D-Allose; B – Ofloxacin; C – Oxolinic acid. Statistical values for each of these individual graphs can be found in Table 2. These traces show an inverse response when compared to figure 7, where conversion by $\phi 24_B$::Kan has a negative effect on the respiration of MC1061.

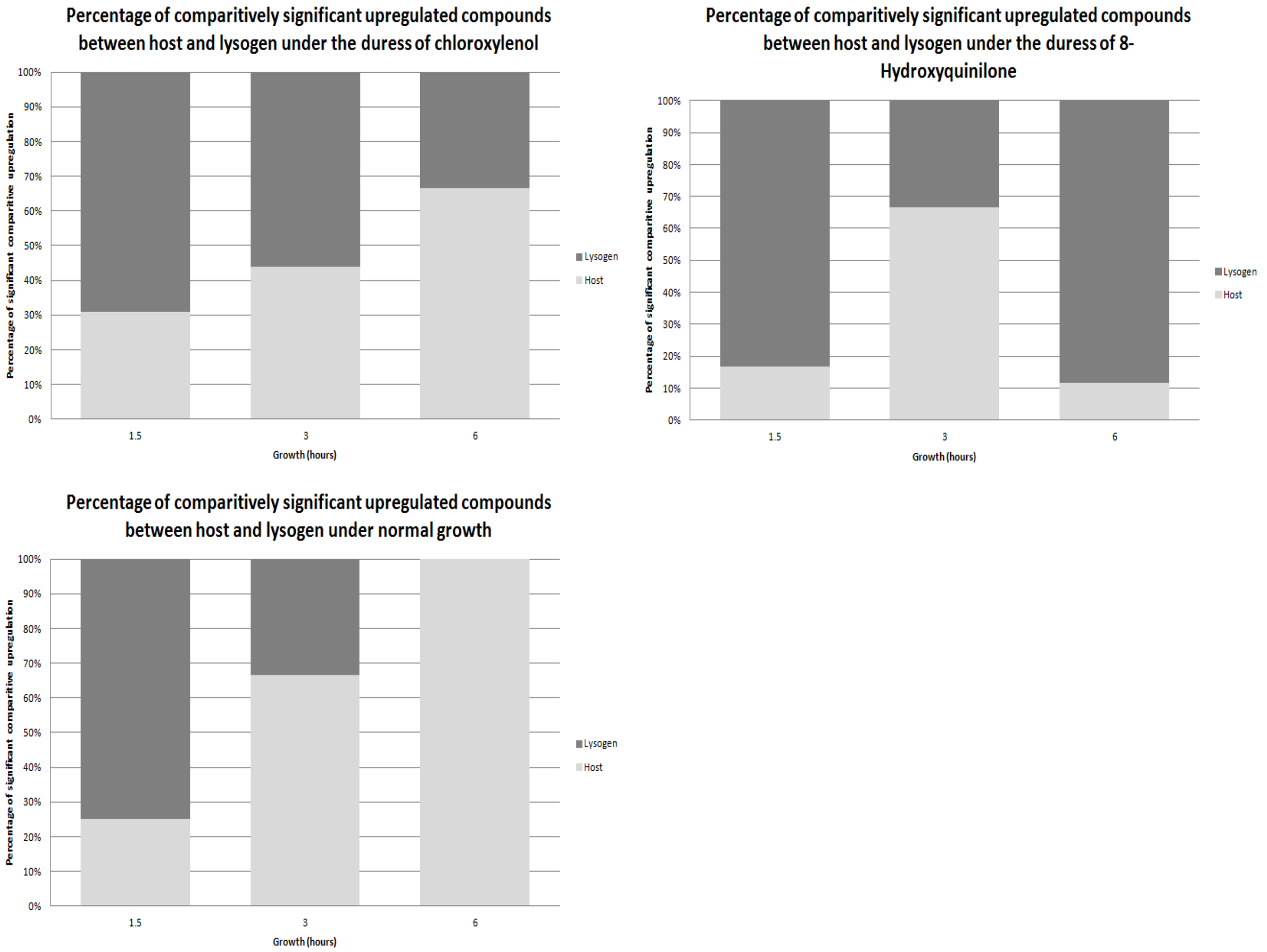

SI Figure 3 Percentage differences of metabolites present in MC1061 and $\phi 24_B$, where incidence is significantly higher (P value <0.05), sampled during growth, under test 8-hydroxyquinoline and chloroxylenol

SI Table 3 Putative metabolite identities and statistics:

Compound/Retention time/Mz		Putative ID	mass error ppm	Fragment peak matches	No. of fragments matching top 5 reference fragments	Adduct of compound	Formula of compound	isotope similarity	Anova (*P*)	Max Fold Change	Upregulated
1	1.71_217.8647m/z	N/A	N/A	N/A	N/A	N/A	N/A	N/A	0.00163439	1.191	Host
2	9.37_173.0806m/z	2-PROpylglutanic acid	-7.5	7	0	M-H	C8H14O4	95.03	0.00063323	1.452	Phage
3	11.62_742.1533n	N/A	N/A	N/A	N/A	N/A	N/A	N/A	0.03458018	1.364	Phage
4	11.80_293.1760m/z	Myrsinone	0.59	6	2	M-H	C17H26O4	95.9	0.00002419	1.201	Phage
5	12.83_384.1933n	ARMI	-1.1	10	4	M+NA or M+K or M+H	C23H28O5	93.42	0.00000001	1.892	Host
6	10.12_273.1951n	HEPTAN	3.82	5	0	M+H or M+Na	C14H27NO4	96.95	0.00056996	1.182	Phage
7	10.51_287.2924n	N/A	N/A	N/A	N/A	N/A	N/A	N/A	0.00008937	1.230	Phage
8	11.13_772.3460n	N/A	N/A	N/A	N/A	N/A	N/A	N/A	0.00005400	1.253	Phage
9	6.62_192.1364n	N/A	N/A	N/A	N/A	N/A	N/A	N/A	0.00000048	1.341	Phage
10	9.71_176.1808n	CANAV	510	0	0	M+Na or M+K	C5H12N4O3	95.5	0.00010806	1.639	Host
11	1.85_161.0810m/z	Ethyl	1.28	4	0	M+H	C7H12O4	91.78	0.00120174	1.161	Phage
12	11.96_420.3836m/z	EUROCOYL	-0.1	3	2	M+ACN+H	C25H46O2	95.49	0.00000010	1.428	Phage

13	2.14_220.1546m/z	MIGLUSTAT	1.1	12	3	M+H	C10H21NO4	31.1	0.00007761	1.136	Host
14	8.93_277.1291m/z	Pentoxifylline	-5.4	1	0	M-H	C13H18N4O3	88.77	0.03615228	1.074	Host
15	6.00_174.0396m/z	FAPy-Adenine	-1	2	0	M+Na-2H	C5H7N5O	80.57	0.00190075	1.426	Host
16	12.17_381.0798n	4-{[(2,6-dichlorophenyl)carbonyl]amino}-N-piperidin-4-yl-1H-pyrazole-3-carboxamide	10.3	3	1	M+H or M+Na or M+K	C16H17Cl2N5O2	90.83	0.02445850	1.060	Phage
5	12.83_384.1933n	ARM	-1	10	4	M+NA or M+K or M+H	C23H28O5	93.52	0.00672773	1.280	Host
17	11.93_252.1728n	Epioxylubimin	1.05	25	2	M+H OR M+Na	C15H24O3	93.23	0.00453567	1.142	Host
18	11.79_295.1910m/z	9-DECENO	0.62	5	0	M+K	C15H30NO2	96.92	0.01428060	1.091	Phage
18	11.79_295.1910m/z	Gingerol	1.99	10	3	M+H	C17H26O4	96.92			
19	11.95_233.1540m/z	Turmeronol B	1.53	9	2	M+H	C15H20O2	75.12	0.00010829	1.779	Host
19	11.95_233.1540m/z	1,3,11(13)-EUD	1.53	9	1	M+H	C15H20O2	75.12			
20	6.11_192.0995m/z	Epsilon-heptenoic acid	0.17	3	0	M+ACN+Na	C7H12O2	92.2	0.04028174	1.084	Phage
12	11.97_420.3837m/z	Erucoylacetone	0.2	3	2	M+ACN+H	C25H46O2	95.73	0.00991001	1.259	Phage
21	11.12_370.0693m/z	Citbrasine	1.6	8	3	M+K	C17H17NO6	95.32	0.00070557	1.109	Phage
22	10.25_272.2594m/z	hexadecanoic acid	3.53	16	4	M+H	C16H33NO2	94.86	0.01076165	1.080	Phage
23	9.63_286.1762m/z	N/A	N/A	N/A	N/A	N/A	N/A	N/A	0.00031671	2.413	Host

24	7.61_311.1832m/z	n5-Pan	-38	0	0	M+Na	C14H28N2O4	84.31	0.00069227	2.311	Host
25	11.12_368.0724m/z	Alpha-Methylene Adenosine Monophosphate	-1.9	7	2	M+Na	C11H16N5O6P	95.36	0.00056106	1.090	Phage
26	10.78_286.2846m/z	N/A	N/A	N/A	N/A	N/A	N/A	N/A	0.01460937	1.164	Phage
27	10.68_288.2895m/z	Sphinganine	-0.6	15	5	M+H	C17H37NO2	97.63	0.00441829	1.135	Phage
2A	8.37_174.0879n	Ethyladipic acid or 2-Propylglutaric acid	-7.7	8	1	M-H, or M+Na-2H	C8H14O4	95.79	0.00478386	1.847	Host
28	10.79_278.9872m/z	2-Keto-3-deoxy-6-phosphogluconic acid	-6.1	8	1	M+Na-2H	C6H11O9P	92.2	0.02553743	1.086	Host
28 (2nd ID)	10.79_278.9872m/z	ETHOXZ	-3	2	0	M+Na-2H	C9H10N2O3S2	81.06			
29	10.93_256.9850m/z	6-phosphono	-85	5	0	M-H	C6H11O9P	94.62	0.03357850	1.084	Host
30	11.17_346.9947m/z	2-(ALPHA	-126	5	0	M-H	C9H17O12P	88.02	0.01381940	1.101	Host
31	9.88_378.1772m/z	3,3-DIMETHYL	93.2	2	0	M-H	C16H21N5O6	88.13	0.00000000	1.621	Phage
32	2.82_236.0782m/z	8-[(Amino	-1	3	0	M-H	C9H19NO2S2	85.57	0.00000008	1.591	Host
32	2.82_236.0782m/z	N-(1-Deoxy-1-fructosyl)glycine	2.69	5	0	M-H	C8H15NO7	94.85			
33	5.28_309.1189m/z	Imazam	-11	1	0	M+Na-2H	C16H20N2O3	86.45	0.03600555	1.390	Phage
33	5.28_309.1189m/z	Desloratad	8.12	0	0	M-H	C19H19ClN2	63.92			
34	1.42_257.0776m/z	Imidazoleacetic acid riboside	-1.2	7	1	M-H	C10H14N2O6	94.13	0.03095166	1.094	Host
35	1.93_212.9722n	2 AMINO or L ASPARTYL	-149	0	0	M+H or M+ACN+H or M+Na	C4H8NO7P	94.5	0.00477583	1.119	Host

36	10.32_268.1505n	Isoleucyl-Histodine or Histidinyl-isoleucine or Histidinyl-Leucine or Leucyl-Histodine	-12	7	0	M+H or M+Na	C12H20N4O3	92.25	0.00000002	1.607	Phage
37	1.83_259.0926m/z	5-Methyluridine	0.45	7	2	M+H	C10H14N2O6	96.5	0.04657029	1.058	Host
38	9.29_251.1647m/z	1HYDROXY	2.19	7	0	M+H	C15H22O3	95.8	0.00000007	1.618	Phage
39	13.36_1020.0887n	N/A	N/A	N/A	N/A	N/A	N/A	N/A	0.00020510	1.252	Phage
40	9.13_514.2625n	GLYCINO	11.3	2	1	M-H or M+Na-2H	C29H38O8	84.79	0.00259487	1.122	Phage
41	13.52_297.1529m/z	GRAVEL	11	4	1	M-H	C19H22O3	94.99	0.00674144	1.263	Host
42	11.96_565.0481m/z	URIDINE	0.59	7	0	M-H	C15H24N2O17P2	83.68	0.01779212	1.070	Phage
43	12.68_733.0280m/z	N/A	N/A	N/A	N/A	N/A	N/A	N/A	0.00924811	1.111	Phage
44	2.50_175.1443m/z	N-Dimethyl-lysine	1.08	6	0	M+H	C8H18N2O2	97.67	0.03036812	1.572	Phage
45	1.98_186.0128n	3-PHOSPHO	107	4	0	M-H or M+Na-2H	C3H7O7P	97.91	0.00021209	1.152	Host
4	11.82_293.1760m/z	Myrsinone	0.71	6	2	M-H	C17H26O4	95.65	0.03289389	1.068	Host
46	10.85_179.1065m/z	3 POSS ID'S	147	0	0	M-H	C6H14NO5	92.2	0.03020868	1.118	Host
47	5.93_252.1575n	2-(2-{2-[2-(2-Methoxy-Ethoxy)-Ethoxy]-Ethoxy}-Ethoxy)-Ethanol	0.86	5	0	M+H or M+Na	C11H24O6	92.56	0.03127143	1.085	Host
48	8.16_286.1652m/z	POLYETHYLENE	1.3	8	1	M+ACN+H	C12H20O5	88.26	0.00000068	1.304	Phage
49	7.75_203.1280m/z	SEBACIC ACID	1.31	5	0	M+H	C10H18O4	88.75	0.00001700	1.226	Phage
50	4.72_1100.3683n	N/A	N/A	N/A	N/A	N/A	N/A	N/A	0.00000026	1.560	Phage

51	9.47_454.2408n	Ethyl Cellulose	-1.3	4	0	M-H or M+Na-2H	C20H38O11	92.02	0.00000026	1.560	Phage
39	13.36_1020.0885n	N/A	N/A	N/A	N/A	N/A	N/A	N/A	0.01735946	1.051	Phage
52	1.42_365.0428n	N/A	N/A	N/A	N/A	N/A	N/A	N/A	0.02345941	1.118	Host
53	7.71_209.0445m/z	3-(3,4-Dihydroxy-5-methoxy)-2-propenoic acid	-4.9	2	0	M-H	C10H10O5	95.49	0.00007118	1.223	Phage
54	10.64_264.9911m/z	RU78299	11.4	5	1	M+Na-2H	C9H9O6P	87.27	0.03671033	1.144	Phage
55	11.05_253.1440m/z	GALACTO	201	2	0	M-H	C9H18O8	97.51	0.02875719	1.052	Host
56	6.45_259.1184m/z	Glycerol tripropanoate	-1	0	0	M-H	C12H20O6	90.27	0.00794597	1.089	Host
15	5.96_174.0395m/z	Diureido-Acetate	0.43	0	0	M-H	C4H7N4O4	81	0.00000763	2.459	Phage
57	9.34_328.1765m/z	N/A	N/A	N/A	N/A	N/A	N/A	N/A	0.00000854	1.634	Phage
58	1.42_459.9496m/z	POLYTHIAZIDE	9.18	3	0	M+Na-2H	C11H13ClF3N3O4S3	58.45	0.00007997	2.680	Phage
59	9.67_297.1586n	3-[(4-AMINO-1-TERT-BUTYL-1H-PYRAZOLO[3,4-D]PYRIMIDIN-3-YL)METHYL]PHENOL	-1.4	5	0	M+H or M+Na	C16H19N5O	92.81	0.00000037	1.913	Phage
60	9.89_242.1505n	N/A	N/A	N/A	N/A	N/A	N/A	N/A	0.00000754	1.899	Phage
61	1.43_289.1277n	Ophthalmic acid	1.31	7	0	M+H or M+Na or M+K	C11H19N3O6	95.35	0.00000120	1.327	Phage
38	9.77_251.1649m/z	N1-ACTEYL-103.12		0	0	M+ACN+Na	C9H21N3O	97.4	0.00003191	2.204	Phage
62	9.29_402.2860m/z	Sphingofungin F	2.62	5	0	M+H	C21H39NO6	88.38	0.00004939	2.350	Phage
63	4.45_843.2912m/z	3-Sialyl Lewis	6.57	14	2	M+Na	C31H52N2O23	94.34	0.04643126	1.100	Phage

64	9.71_269.1755m/z	Capryloylcholine	1.4	5	2	M+K	C13H28NO2	96.32	0.00001729	2.086	Phage
S1	10.26_411.1987m/z	icariside B8	-0.5	11	1	M+Na	C19H32O8	84.89	0.03032644	1.157	Host
S2	10.26_277.1407m/z	1-[(2-Amino-6,9-Dihydro-1h-Purin-6-Yl)Oxy]-3-Methyl-2-Butanol	-0	2	0	M+ACN+H	C10H13N5O2	91.1	0.02763331	1.171	Phage
S3	9.65_389.1827m/z	(-)-11-hyrdoxy-9,10-dihydrojasmonic acid 11-beta-D-glucoside	2.58	4	0	M-H	C18H30O9	83.64	0.04213067	1.095	Phage
S4	10.68_207.1020m/z	tuberonic acid or 12-hydroxyjasmonic acid or epi-4'-hydroxyjasmonic acid	-2.9	8	0	M-H20-H	C12H18O4	94.4	0.02510448	1.105	Phage
S5	10.47_240.1956m/z	RISHITIN	-1	27	1	M+NH4	C14H22O2	93.93	0.03927680	1.804	Phage
S6	11.29_223.1005m/z	1-METHYL-6-PHENYL-1h-IMIDAZOL[4,5-B]PYRIDIN-2-AMINE	7.26	0	0	M-H	C13H12N4	86.21	0.02797408	1.113	Host
S7	1.29_348.8967m/z	N/A	N/A	N/A	N/A	N/A	N/A	N/A	0.04022452	1.273	Host
S8	1.37_178.1075m/z	Pimelic acid	0.56	8	2	M+NH4	C7H12O4	67.66	0.02768047	1.291	Host
S9	1.35_164.0919m/z	3-DEOXY-d-GLUCOSAMINE	0.82	14	0	M+H	C6H13NO4	88.26	0.03943001	1.385	Host
S10	11.63_467.0141m/z	deoxyuridine triphosphate	102	0	0	M-H	C9H15N2O14P3	88.23	0.01819624	1.072	Host
S11	11.65_473.0048m/z	ALLURA RED AC	-10	6	1	M+Na-2H	C18H16N2O8S2	75.97	0.00211876	1.078	Host
S12	12.24_555.0084m/z	N/A	N/A	N/A	N/A	N/A	N/A	N/A	0.00358126	1.082	Host
S13	11.45_455.0141m/z	HALOSULFURON-METHYL	-3.9	2	0	M+Na-2H	C13H15ClN6O7S	63.46	0.01500173	1.064	Host
S14	10.44_252.9904m/z	RU78300	8.8	8	0	M+Na-2H	C8H9O6P	90.34	0.02427095	1.077	Host

S15	10.58_341.0050m/z	1,6-Di-O-Phosphono-D-Allitol (or -mannitol)	1.73	2	0	M-H	C6H16O12P2	94.13	0.00830116	1.079	Host
S16	11.43_469.0292m/z	N/A	N/A	N/A	N/A	N/A	N/A	N/A	0.00229216	1.091	Host

Table details the 80 metabolites that have been deemed significant using CV percentage, chromatogram peaks, statistical analysis and confirmed using MS-MS and fragmentation analysis under antimicrobial challenge of MC1061 and Lysogen. The 16 metabolites (S1-S16) determined to be different metabolites that discriminate between MC1061 and Lysogen under standard laboratory growth conditions. Table lists retention times and Mz from the column. It also includes putative compound ID based from comparison searches against pre-determined databases. It also determines fragment scores of MS-MS and the level of change in abundance whether associated with MC1061 (host) or the Lysogen (phage).

SI Table 4. Statistics for compound ID's (sustained with reputable MSMS fragmentation) related to known bacterial pathways

m/z	ID	Mass error (ppm)	Adduct	Formula	Anova (p)	Max Fold Change	Up regulated
174.0396	FAPy-Adenine	0.69	M-H	C4H7N4O4	0.0019	1.43	Naïve Host
272.2594	hexadecanoic acid	3.53	M+H	C16H33NO2	0.01	1.08	Lysogen
288.2895	Sphinganine	-0.64	M+H	C17H37NO2	0.0044	1.13	Lysogen
259.0926	5-Methyluridine	0.45	M+H	C10H14N2O6	0.0466	1.06	Naïve Host
289.1277 n	Ophthalmic acid	1.31	M+H or M+Na or M+K	C11H19N3O6	0.0000012	1.33	Lysogen
178.1075	Pimelic acid	0.56	M+NH4	C7H12O4	0.0277	1.29	Lysogen

SI Table 5. PLS-DA statistics

PLS-DA Standard conditions				
Component	Eigenvalue	R2Y(cum)	Q2	Q2(cum)
1	10.6	0.262	-0.556	-0.1
2	4.06	0.847	0.755	0.73
PLS-DA chloroxylenol conditions				
Component	Eigenvalue	R2Y(cum)	Q2	Q2(cum)
1	4	0.923	0.802	0.802
2	2.71	0.981	0.405	0.882
PLS-DA 8-hydroxyquinoline conditions				
Component	Eigenvalue	R2Y(cum)	Q2	Q2(cum)
1	3.84	0.89	0.74	0.74
2	3.75	0.967	0.533	0.879

Supplementary Information (SI) - Methods

Bacterial phenotypic microarray

The panel plates used for this study included Biolog plates PM 1-20, which include a plethora of both metabolic and toxicological effectors. The various plats test: PM 1, 2a-Respiration and metabolism of different carbon sources; PM3B - respiration on different Nitrogen sources; PM4A -utilisation of different phosphorous and sulphur sources; PM5 - utilisation of other nutrient supplements including amino acids; PM 6,7,8 i- utilisation of a range of peptide Nitrogen sources; PM 9 –impact of osmolytes on respiration; PM10 - effect of pH on respiration; PM11C, 12B, 13B, 14A, 15B, 16A, 17A, 18C, 19 and 20B – respiration impacted by a wide range of chemical compounds including antibiotics.
